# National Antimicrobial Stewardship Performance and Antimicrobial Use in Saudi Ministry of Health Hospitals: A Four-Year Multicenter Surveillance Analysis, 2021–2024

**DOI:** 10.3390/antibiotics15080753

**Published:** 2026-08-04

**Authors:** Saleh Alghamdi, Mohammad Algarni, Alaa Mutlaq, Reema Almuraybidh, Nawal Alfardus, Sumaiah Aljudaibi, Amnah Aljaffar, Ahmed Alghamdi, Salman Alghamdi, Abdullah S. Alshammari, Abdulhakim A. Alzahrani, Bassant Mohamed Barakat, Mohammad A. Albanghali

**Affiliations:** 1 Department of Clinical Pharmacy, Faculty of Pharmacy, Al-Baha University, Al-Baha 65779, Saudi Arabia; saleh.alghamdi@bu.edu.sa (S.A.); bbarakat@bu.edu.sa (B.M.B.); 2 General Administration of Pharmaceutical Care, Ministry of Health, Riyadh 11451, Saudi Arabia; asmutlaq@moh.gov.sa (A.M.); ralmuraybidh@moh.gov.sa (R.A.); nalfardus@moh.gov.sa (N.A.); 3 Hospital Support Operations Centre, Ministry of Health, Riyadh 11176, Saudi Arabia; saljudiabi@moh.gov.sa; 4 Department of Pharmaceutical Care, Dammam Medical Complex, Eastern Health Cluster, Dammam 32253, Saudi Arabia; amaljaffar@moh.gov.sa; 5 Department of Clinical Pharmacy, College of Pharmacy, King Saud University, Riyadh 11451, Saudi Arabia; aanahi@ksu.edu.sa; 6 Department of Pharmaceutical Care, Almikwah Hospital, Al-Baha Health Cluster, Ministry of Health, Al-Baha 65784, Saudi Arabia; samualghamdi@moh.gov.sa; 7 Pharmaceutical Practices Department, College of Pharmacy, Umm Al-Qura University, Makkah 24237, Saudi Arabia; asshammari@uqu.edu.sa; 8 Department of Pharmaceutical Care, King Fahad Hospital, Al-Baha Health Cluster, Ministry of Health, Al-Baha 57911, Saudi Arabia; aalzahrani116@moh.gov.sa; 9 Department of Public Health, Faculty of Applied Medical Sciences, Al-Baha University, Al-Baha 57911, Saudi Arabia; mohammad.aref@bu.edu.sa

**Keywords:** antimicrobial stewardship, Saudi Arabia, performance indicators, protocol adherence, restricted antibiotic dispensing, physician acceptance, therapeutic drug monitoring

## Abstract

**Background/Objectives:** Antimicrobial stewardship programmes (ASPs) are essential for improving antimicrobial use and limiting the emergence of antimicrobial resistance. Although national ASP key performance indicators (KPIs) have been implemented across Saudi Ministry of Health (MOH) hospitals, large-scale evaluations of programme performance remain limited. This study evaluated national ASP performance across MOH hospitals from 2021 to 2024 using formal KPIs and complementary stewardship measures. **Methods:** This retrospective multicentre surveillance analysis included 124,452 antimicrobial prescription reviews from 96 Saudi MOH hospitals actively reporting stewardship activities. Hospitals were classified according to ASP capacity as category A, B, or C. The evaluated KPIs included protocol adherence, restricted-antibiotic dispensing compliance, active intervention rate, annual trends in optimization interventions, and physician acceptance of ASP recommendations. Secondary assessments included appropriateness of therapy management, therapeutic drug monitoring (TDM) for vancomycin and aminoglycosides, and hospital-level antimicrobial use expressed as defined daily doses per 100 available bed-days. Multivariable logistic regression was used to identify factors associated with guideline non-adherence and physician non-acceptance. **Results:** Overall protocol adherence was 87.1%, exceeding the national target of 80%, while the active intervention rate was 64.6%, exceeding the 60% benchmark. Restricted-antibiotic dispensing compliance reached 87.6% but remained below the 100% target. Physician acceptance of ASP recommendations was high at 93.0%, although it did not achieve the national target of 98%. The optimization-intervention KPI was not met because de-escalation, antibiotic discontinuation, and intravenous-to-oral conversion did not demonstrate a sustained annual increase. Appropriate ongoing therapy management was documented in 68.3% of cases with available data, whereas TDM was requested in 44.5% of eligible vancomycin or aminoglycoside cases. Antimicrobial use varied across years and hospital categories, with higher monitored broad-spectrum and reserve-agent use in higher-capacity hospitals. Lower ASP capacity, non-critical care settings, and combination therapy were consistently associated with less favourable stewardship outcomes. **Conclusions:** Saudi MOH hospitals actively reporting stewardship activities demonstrated favourable performance in protocol adherence, intervention activity, and physician acceptance. However, persistent gaps in restricted-antibiotic dispensing, optimization interventions, TDM, and inter-hospital variation indicate the need for continued strengthening of ASP infrastructure, monitoring, and feedback systems. Future research should link stewardship indicators with clinical outcomes, antimicrobial resistance trends, and patient safety.

## 1. Introduction

Antibacterial innovation remains limited despite the growing clinical need for effective agents against resistant infections [1]. The World Health Organization’s 2020 antibacterial pipeline analysis reported that 11 new antibacterial agents had been approved since 2017 and that only two, meropenem–vaborbactam and Lefamulin, met at least one WHO criterion for innovation [2]. This constrained pipeline reinforces the importance of preserving the effectiveness of existing antimicrobial therapies [1].

Antimicrobial agents differ from many other medicines because their use has consequences for both the treated patient and society. Antimicrobial exposure creates selective pressure that promotes the emergence of drug-resistant bacteria [3]. The World Health Assembly’s endorsement of the Global Action Plan on antimicrobial resistance (AMR) in 2015 [4] and the UN General Assembly’s political declaration on AMR in 2016 [5] formalized AMR as a major global public health threat. In 2019, bacterial AMR was associated with an estimated 4.95 million deaths worldwide, including 1.27 million deaths directly attributable to resistance [6]. Earlier projections suggested that, without effective intervention, AMR could impose a severe long-term health and economic burden [7].

Hospital antimicrobial use remains a major stewardship target. Previous work has documented substantial inappropriate or unnecessary inpatient antibiotic use [8]. Such use may be driven by diagnostic uncertainty, delayed diagnostic information, system-level incentives, and gaps in prescriber knowledge [9,10,11,12]. Social and organizational norms may further shape prescribing behaviours and contribute to unnecessary reliance on broad-spectrum or reserve agents [13,14,15].

In Saudi Arabia, an estimated 2260 deaths were attributable to antimicrobial resistance, and 8620 deaths were associated with antimicrobial resistance in 2021, underscoring the national burden of resistant infections [16]. A point-prevalence survey in Makkah-region public hospitals found that 61.9% of 710 hospitalized patients were receiving at least one antimicrobial agent [17]. These findings reinforce the need for large-scale evaluation of stewardship performance within the Saudi MOH hospital system.

Given the constrained antibacterial pipeline and the continuing emergence of resistance, ASPs are central to optimizing the use of existing antimicrobial agents [18,19]. Antimicrobial stewardship comprises coordinated actions designed to improve and monitor appropriate antimicrobial use, including selection, dosing, duration, and route of administration. These measures are intended to improve patient care, minimize avoidable harms, reduce antimicrobial resistance pressure, and support efficient resource use [20,21,22,23].

Hospital-based ASP implementation has been associated with reductions in antimicrobial use and use of restricted agents [24]. Prescriber engagement is also essential: the practical value of ASP recommendations depends in part on whether treating clinicians accept and implement them [25,26]. A national survey of 247 Saudi MOH hospitals reported a 54% response rate, with only 26% of responding hospitals indicating that ASPs had been implemented [27]. This highlights the need for contemporary, large-scale evaluation of ASP performance after the subsequent expansion of stewardship activity within MOH hospitals.

Despite increasing policy attention, large-scale multicentre evidence describing ASP performance across Saudi MOH hospitals using standardized quantitative indicators remains limited. There is a need to evaluate protocol adherence, restricted-antibiotic dispensing compliance, stewardship intervention activity, physician acceptance of ASP recommendations, therapeutic drug monitoring (TDM), antimicrobial use, and factors associated with suboptimal performance. Therefore, this retrospective multicentre surveillance analysis aimed to evaluate ASP performance across 96 Saudi MOH hospitals that were actively reporting ASP activities to the MOH during the study period from 2021 to 2024. Specifically, the study aimed to: (1) assess national ASP key performance indicators, including protocol adherence, restricted-antibiotic dispensing compliance, active intervention rate, stewardship-driven optimization interventions, and physician acceptance of ASP recommendations; (2) examine secondary non-KPI assessments, including appropriate therapy management, TDM practices for vancomycin and aminoglycosides and their relationship with dose adjustment, and hospital-level use of monitored broad-spectrum antimicrobial agents; and (3) identify independent predictors of guideline non-adherence and physician non-acceptance of ASP recommendations.

## 2. Results

### 2.1. Demographic and Institutional Characteristics of the Study Population

The antimicrobial stewardship surveillance dataset comprised 124,452 patient records collected across Saudi health clusters from 2021 to 2024. Most records were from 2023 (38.7%), followed by 2024 (21.4%), 2022 (20.7%) and 2021 (19.2%). Hospital categories re-flected the level of ASP infrastructure and available resources. Category A hospitals had full ASP implementation, including a complete core team with infectious-disease physi-cian/consultant involvement, specialized clinical pharmacy support, diagnostic tools, and microbiology/laboratory support. Category B hospitals had partial ASP core-team availa-bility, with either infectious-disease physician or clinical pharmacist involvement and es-sential diagnostic and laboratory resources, primarily using pre-authorisation strategies for restricted antibiotics. Category C hospitals had limited ASP resources, no dedicated ASP team, and relied mainly on a trained pharmacist to monitor protocol adherence, dose optimization, and documentation of stewardship interventions. Hospital category was available for 119,178 records (95.8%); among these, category C accounted for the largest share (40.2%), followed by category B (33.3%) and category A (26.5%). Cases were distributed across the five official regions of Saudi Arabia, with the largest shares from the Western (27.8%) and Central (24.3%) regions and smaller shares from the Southern (20.1%), Northern (14.7%), and Eastern (13.1%) regions. Substantial cluster-level variation within each region is shown in Table 1. Males represented 55.8% of records. The largest age group was ≥65 years (25.4%), while 18–24 years comprised the smallest proportion (5.8%) (Table 1).

### 2.2. Clinical Characteristics, Infection Types, and Antimicrobial Resistance Patterns

A total of 124,452 encounters were included. Most cases were managed in non-critical care wards (58.6%), while 41.4% occurred in critical care units. Pneumonia and respiratory infections were the most frequent syndrome (36.2%), followed by bloodstream and systemic infections (23.5%), other infectious conditions (13.6%), and skin and soft tissue infections (11.9%). Abdominal and gastrointestinal (8.8%) and genitourinary and sexually transmitted infections (7.9%) were less common, with all remaining categories each accounting for ≤3.9% of cases (Table 2). Documented laboratory-confirmed resistance indicators were infrequently recorded at the case level: ESBL was documented in 1326 cases (1.1%), MDR in 1117 cases (0.9%), CRE in 917 cases (0.7%), OXA-48 in 102 cases (0.1%), NDM in 107 cases (0.1%), VRE in 69 cases (0.06%, rounded to 0.1%), and XDR in 517 cases (0.4%) (Table 2). These percentages represent the frequency of documented resistance indicators among ASP-reviewed antibiotic-treated inpatient cases and should not be interpreted as isolate-level antimicrobial resistance prevalence. Empiric therapy was the most frequent indication for antibiotics (61.1%), followed by culture-guided therapy (25.7%) and surgical prophylaxis (13.2%). Regarding prescribing intensity, monotherapy predominated (85.7%), while combination therapy was used in 14.3% of cases (Table 2).

### 2.3. Antimicrobial Stewardship Programme (ASP) Review and Interventions Overview

Among all reviewed prescriptions, 71,413 (57.4%) were documented as prescribed according to MOH or hospital protocols, 10,582 (8.5%) were not, and 42,457 (34.1%) were classified as having a diagnosis outside available protocol indications, that is, a recorded diagnosis or indication that could not be assessed against any MOH or institutional antimicrobial protocol in the surveillance dataset. A full characterization of the syndrome distribution, leading individual diagnoses, and indications for these 42,457 cases is provided in Supplementary Table S1. Bloodstream and systemic infections were the most frequent primary syndrome (29.4%), followed by other infectious conditions (20.6%), pneumonia and respiratory infections (14.1%), skin and soft tissue infections (12.3%), and abdominal and gastrointestinal infections (7.9%). At the diagnosis level, surgical prophylaxis was the single most frequent individual diagnosis (20.5%), followed by sepsis (14.6%) and septic shock (5.7%); by indication, 54.7% of outside-protocol prescriptions were empirical, 24.0% culture-guided, and 21.4% surgical prophylaxis. These patterns reflect the absence of dedicated national protocols for sepsis and bacteraemia and the fact that many surgical indications fell outside the scope of the current surgical site-infection protocol; both represent priority areas for future protocol expansion.

The appropriateness of the initial antimicrobial regimen, encompassing drug, dose, route, and duration, was rated appropriate in 69.2% (n = 86,118) of prescriptions and inappropriate in 30.8% (n = 38,334) (Table 3). Ongoing therapy management was rated appropriate in 68.3% (n = 52,958) of cases with available management data, whereas 31.7% (n = 24,630) warranted a stewardship intervention. Therapeutic drug monitoring for vancomycin or aminoglycosides was requested in 44.5% (n = 8990) of eligible cases, not requested in 53.7% (n = 10,841), and not required in 1.8% (n = 366). Physician acceptance of proposed ASP recommendations was documented in 63.3% (n = 78,787) of reviewed cases, non-acceptance in 4.8% (n = 5960), and no active recommendation was required in the remaining 31.9% (n = 39,705); a comprehensive analysis of physician acceptance stratified by health cluster, ward class, and hospital category is presented in Section 2.8, where performance is assessed against the national target of 98.0%.

To characterize how these overall indicators varied by hospital ASP capacity, the same outcomes were stratified across the three hospital categories using the 119,178 records with recorded hospital category (category A, n = 31,610; category B, n = 39,654; category C, n = 47,914). Protocol adherence differed significantly across categories (*p* < 0.001): the proportion prescribed according to protocol was highest in category A (62.5%), followed by category C (57.5%) and category B (53.5%); non-adherence was lowest in category A (5.5%) and higher in categories B and C (9.3% and 9.0%, respectively); and prescriptions with a diagnosis outside available protocol indications were most frequent in category B (37.2%). Appropriateness of the initial regimen was highest in category C (73.8%), intermediate in category A (70.0%), and lowest in category B (64.8%) (*p* < 0.001). Appropriate ongoing therapy management was documented in 62.3% of category A cases, 68.6% of category B cases, and 74.2% of category C cases (*p* < 0.001). These unadjusted differences should be interpreted cautiously because they may reflect differences in case complexity, documentation practices, and the intensity with which ASP teams identified and recorded optimization opportunities, rather than true differences in prescribing quality. Requesting of therapeutic drug monitoring among eligible cases was highest in category C (50.2%), followed by category B (44.1%) and category A (41.4%) (*p* < 0.001). Physician acceptance of ASP recommendations, restricted to cases with an active recommendation, was highest in category A (96.4%), followed by category B (92.3%) and category C (91.2%) (*p* < 0.001). Full stratified counts, percentages, and chi-square *p*-values are presented in Table 3.

### 2.4. KPI 1: Protocol Adherence Rate (Target: >80%)

Protocol adherence was evaluated among 81,995 protocol-eligible prescriptions, after excluding 42,457 prescriptions classified as outside available protocol indications (Table 3). Among eligible prescriptions, overall protocol adherence was 87.1% (71,413/81,995), exceeding the national KPI 1 target of 80%. Annual adherence rates also exceeded the national target throughout the study period, peaking at 92.0% in 2022, followed by 87.9% in 2021, declining to 83.0% in 2023, and partially recovering to 85.4% in 2024 (*p* < 0.001) (Table 4). Accordingly, KPI 1 was achieved at the aggregate level and in each study year, although the declining trajectory observed between 2022 and 2023 warrants continued monitoring. However, because 34.1% of all reviewed prescriptions were outside available protocol indications, KPI 1 should be interpreted as adherence among prescriptions with an applicable protocol, rather than as a measure of protocol coverage or overall appropriateness across all antimicrobial use.

Hospital category demonstrated a significant association with adherence (Table 4), with the highest rates observed in category A hospitals (91.9%), compared with category B (85.1%) and category C (86.5%) (*p* < 0.001), suggesting a positive impact of fully established ASP infrastructure. Substantial inter-cluster variability was evident, ranging from 68.1% in Hafar AlBatin to 96.0% in Al-Jouf. Similarly, adherence was higher in critical care wards (89.1%) than in non-critical care settings (85.6%) (*p* < 0.001).

At the clinical level, adherence varied by infection syndrome and prescribing characteristics (Table 4). The highest adherence was observed for central nervous system infections (90.2%), pneumonia and respiratory infections (88.9%), and bloodstream and systemic infections (88.8%), whereas lower adherence was noted for other infectious conditions (79.6%). With respect to indication for antibiotics, culture-guided therapy demonstrated the highest adherence (90.7%), compared with empiric therapy (86.2%) and surgical prophylaxis (83.0%) (*p* < 0.001). Monotherapy was associated with higher adherence than combination therapy (88.2% vs. 81.4%; *p* < 0.001) (Table 4).

### 2.5. KPI 2: Restricted-Antibiotic Dispensing Compliance (Target: 100%)

Restricted-antibiotic dispensing compliance was evaluated among category A and B hospitals only (n = 32,676) (Table 5). The overall compliance rate was 87.6%, falling short of the national KPI 2 target of 100% by 12.4 percentage points. Compliance was significantly higher in category A hospitals (90.7%) compared with category B hospitals (85.0%) (*p* < 0.001). Temporal trends demonstrated variation across the study years (*p* < 0.001), with compliance highest in 2024 (91.4%) and 2022 (90.7%), followed by 2021 (87.6%), and lowest in 2023 (84.8%). Marked variability across health clusters was observed (Table 5), with compliance ranging from 42.5% in Hafar AlBatin to 97.7% in Makkah, followed closely by Jazan (97.2%) and Hail (96.6%). Clinical setting influenced compliance, with higher rates in critical care wards (89.7%) compared with non-critical care wards (84.9%) (*p* < 0.001). Infection-specific compliance patterns broadly mirrored those observed for guideline adherence. The highest compliance was reported for bloodstream and systemic infections (90.3%) and pneumonia and respiratory infections (89.1%), whereas substantially lower compliance was observed in other infectious conditions (62.1%) (*p* < 0.001). Culture-guided therapy demonstrated the highest compliance (92.4%), followed by empiric therapy (85.5%), while surgical prophylaxis showed notably lower compliance (66.5%) (*p* < 0.001). Similarly, monotherapy was associated with higher compliance than combination therapy (88.8% vs. 80.9%; *p* < 0.001) (Table 5). Consequently, KPI 2 was not achieved during the study period; the national target of 100% compliance was not reached in any study year or hospital category, with category B hospitals representing the most persistent gap relative to the programme benchmark.

### 2.6. KPI 3: Active Intervention Rate (Target: >60%)

**Figure 1:**
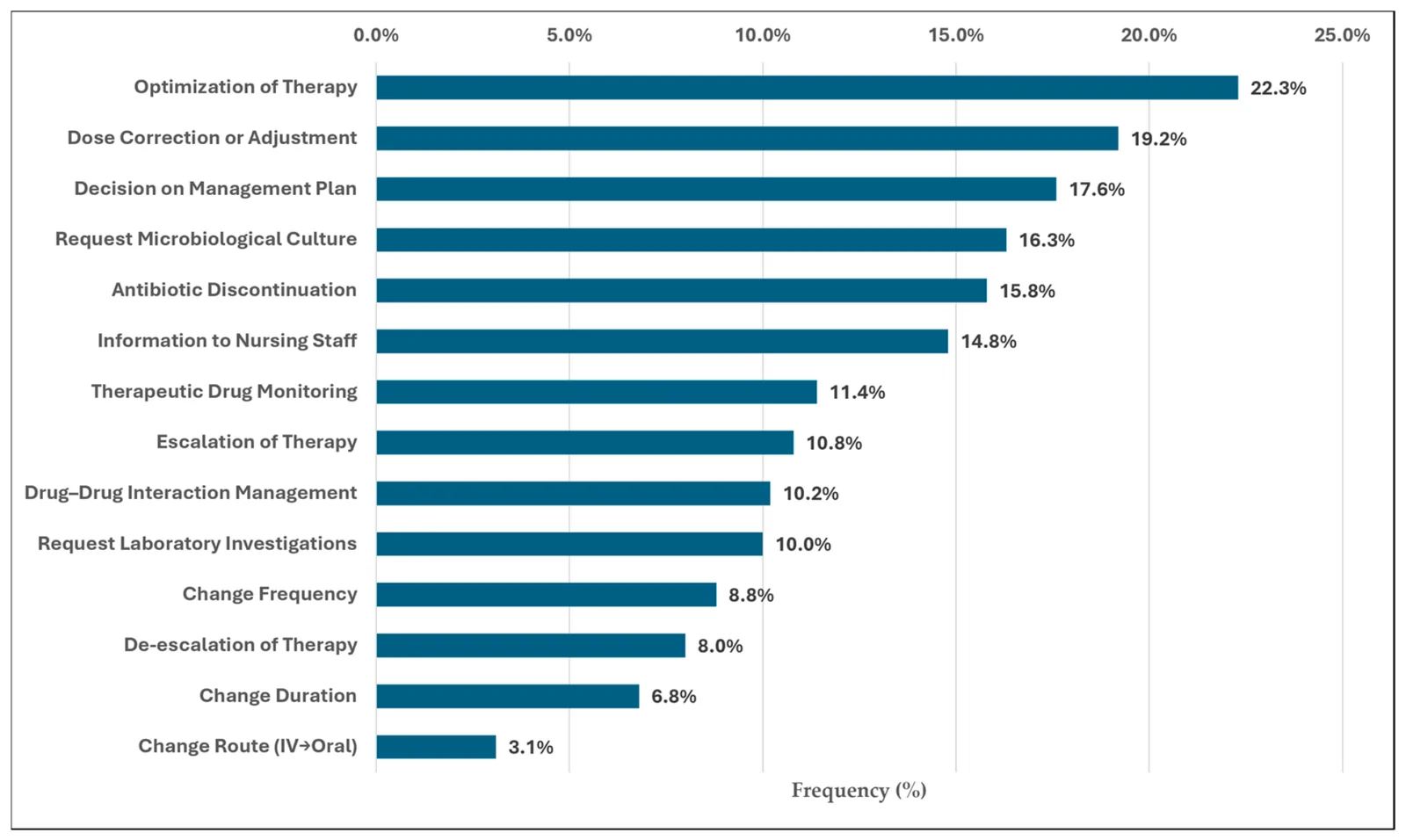
Annual trend in ASP active intervention rate versus cases continued without modification, Saudi MOH hospitals, 2021–2024.

**Figure 2:**
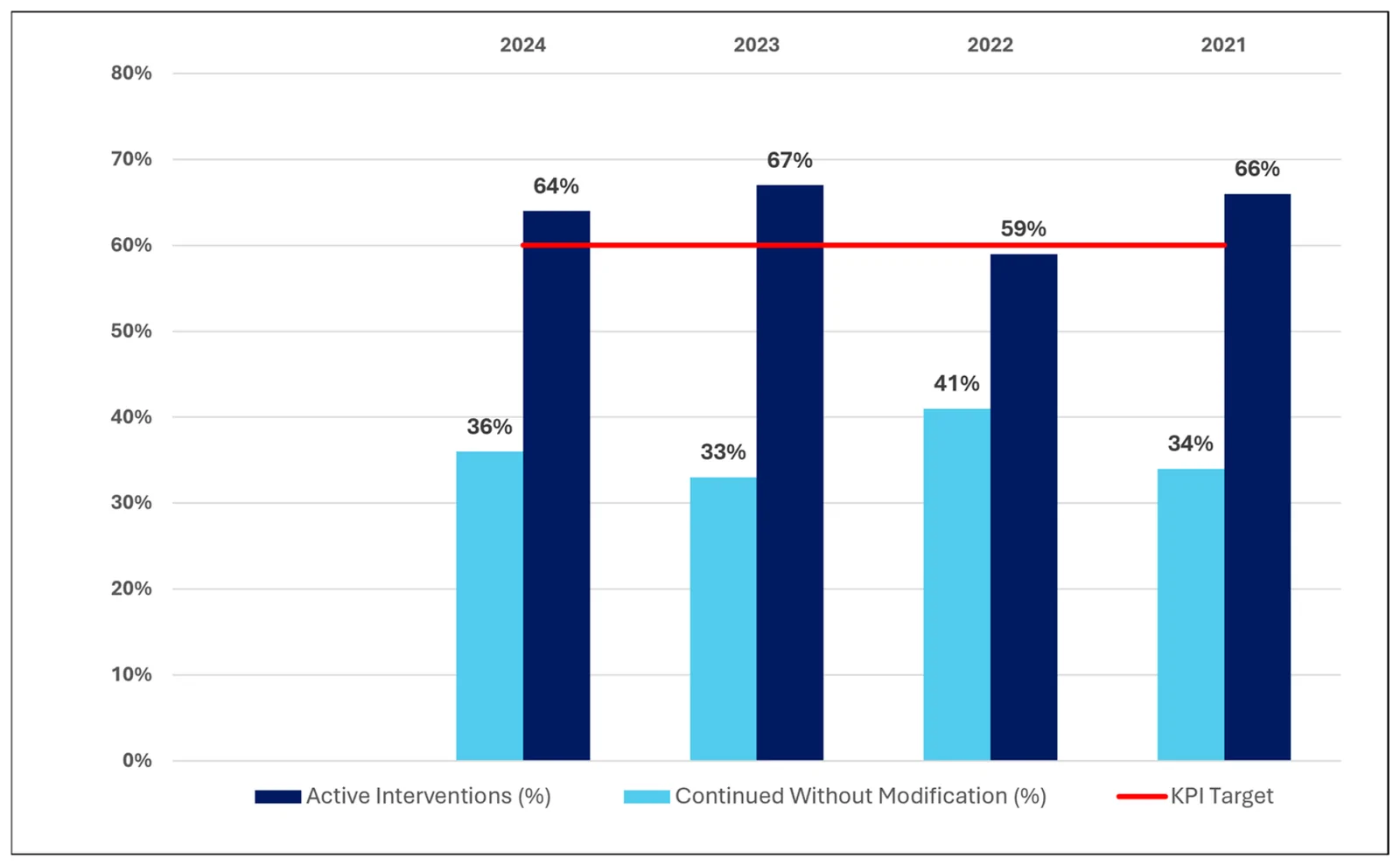
Distribution of ASP intervention types among reviewed antibiotic prescriptions (N = 124,452), Saudi MOH hospitals, 2021–2024. The active intervention rate is defined as the proportion of reviewed cases in which at least one ASP intervention was implemented, including any documented action such as dose adjustment, de-escalation, escalation, discontinuation, optimization, or other stewardship-directed modifications, excluding cases where no change was made. Continued without modification refers to cases in which the antimicrobial regimen was reviewed and deemed appropriate, with no intervention recommended by the ASP team. The red horizontal line indicates the national key performance indicator (KPI) target of 60% for active interventions.

A central performance metric of the national ASP is the proportion of reviewed cases receiving at least one active stewardship intervention, defined as any documented action other than continuation of the existing regimen without modification. The national KPI 3 benchmark requires this rate to exceed 60.0%. Across the full study period, intervention status was documented for 124,347 of 124,452 reviewed cases (105 with missing data); active interventions were recorded in 80,333 of these (64.6%; calculated as [124,347 − 44,014]/124,347 × 100), confirming that KPI 3 was met at the aggregate level. The year-on-year trajectory of this rate over the study period (2021–2024) is presented in Figure 1. The breadth of interventions undertaken by ASP teams across participating hospitals is illustrated in Figure 2 and detailed in Table 6. Optimization of therapy was the most frequently performed intervention (22.3%, n = 27,709), followed by dose correction or adjustment (19.2%, n = 23,925), collaborative decision-making regarding the antimicrobial management plan (17.6%, n = 21,948), the recommendation to obtain microbiological cultures (16.3%, n = 20,274), and antibiotic discontinuation (15.8%, n = 19,601). Interventions of intermediate frequency included the provision of medication administration guidance to nursing staff (14.8%, n = 18,399), requests for laboratory investigations pertinent to antimicrobial follow-up (10.0%, n = 12,504), therapeutic drug monitoring (11.4%, n = 14,241), escalation of antimicrobial therapy in response to clinical deterioration (10.8%, n = 13,482), and measures to address drug–drug interactions (10.2%, n = 12,684). Among the less frequently applied interventions were change in dosing frequency (8.8%, n = 10,969), de-escalation of therapy following clinical improvement or culture guidance (8.0%, n = 9978), modification of treatment duration (6.8%, n = 8512), and intravenous-to-oral route conversion (3.1%, n = 3799) (Table 6; Figure 2). In 35.4% of reviewed cases (n = 44,014), the existing regimen was deemed appropriate and continued without modification (Table 6).

### 2.7. KPI 4: Stewardship-Driven Optimization Interventions (Target: Sustained Increasing Annual Trend)

KPI 4 encompasses three stewardship-driven optimization interventions, de-escalation, antibiotic discontinuation, and intravenous-to-oral route conversion, with programme success defined as a sustained increasing annual trend across all three measures. Assessment of the three stewardship optimization interventions constituting KPI 4 revealed statistically significant annual variation for all three measures (*p* < 0.001) (Table 7). De-escalation remained relatively stable between 2021 and 2023 (8.8%, 8.0%, and 8.7%, respectively) before declining to 6.1% in 2024. Antibiotic discontinuation was the most frequently applied KPI 4 intervention, recorded in 17.6% of cases in 2021 and declining overall to 13.2% in 2024, with a transient stabilization around 16% in 2022–2023. Intravenous-to-oral route conversion was the only intervention to show a net increase across the study period, rising from 2.0% in 2021 to a peak of 3.7% in 2023, before partially declining to 3.2% in 2024. While this represents an overall improvement, the 2024 reversal indicates that gains in IV-to-oral conversion were not sustained, and consistent with the other KPI 4 measures, this intervention did not meet the national benchmark of a sustained increasing annual trend. The combined KPI 4 rate was 23.5% in 2021, which declined to 21.6% in 2022, partially recovered to 23.0% in 2023, and fell to its lowest recorded level of 19.5% in 2024 (*p* < 0.001) (Table 7). The national KPI 4 target of a sustained increasing annual trend was not achieved over the study period, driven primarily by the progressive decline in antibiotic discontinuation rates and only partially offset by improvements in intravenous-to-oral conversion.

### 2.8. KPI 5: Physician Acceptance of ASP Recommendations (Target: >98%)

Physician acceptance of ASP recommendations was evaluated among 84,747 cases in which an active stewardship recommendation was made, excluding the 39,705 cases (31.9%) where no intervention was required. Overall, 93% (n = 78,787) of recommended interventions were accepted by the treating physician, while 7.0% (n = 5960) were declined (Table 3). Against the national programme benchmark, KPI 5 requires a physician acceptance rate exceeding 98.0%; this threshold was not achieved at the aggregate level, in any individual study year, or in any health cluster during the surveillance period. Although acceptance was high in absolute terms, the overall rate fell below the national KPI 5 target of 98.0%, indicating that this benchmark was not met during the study period.

Temporal trends revealed significant variation in acceptance rates across the study years (*p* < 0.001) (Table 8). Acceptance was lowest in 2022 (89.8%), recovering progressively to 93.5% in 2023 and reaching its highest recorded level of 94.8% in 2024. Despite this upward trajectory, no individual year achieved the 98.0% national target.

**Figure 3:**
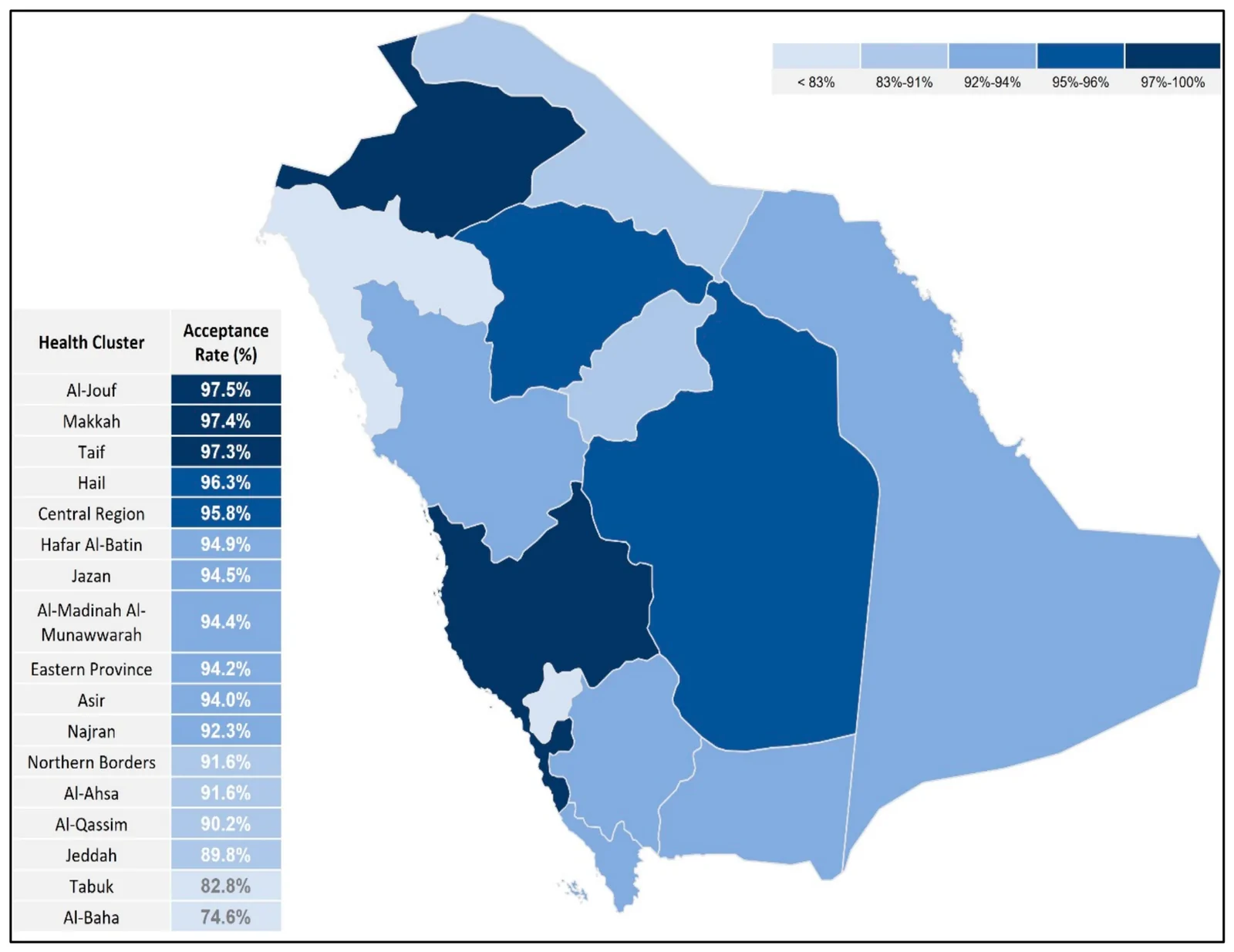
Geographic distribution of physician acceptance rates of ASP interventions across Saudi MOH clusters, 2021–2024 (N = 84,747). Colour intensity reflects the cluster-level acceptance rate, with darker shades indicating higher rates. Acceptance rate is defined as the proportion of ASP-recommended interventions accepted by the treating physician, excluding cases where no intervention was required. The national KPI 5 target is 98.0%; no cluster achieved this threshold during the study period. Al-Jouf recorded the highest acceptance rate (97.5%) and Al-Baha the lowest (74.6%). ASP: Antimicrobial stewardship programme.

Acceptance rates varied significantly by hospital category (*p* < 0.001). Category A hospitals demonstrated the highest rate (96.4%), followed by category B (92.3%) and category C (91.2%) (Table 8). A clinically meaningful difference was observed between ward classes (*p* < 0.001), with acceptance substantially higher in critical care settings (96.2%) compared with non-critical care wards (89.9%) (Table 8). Marked geographic variation in acceptance rates was evident across health clusters (Figure 3). Al-Jouf recorded the highest acceptance rate (97.5%), followed by Makkah (97.4%), Taif (97.3%), and Hail (96.3%). The lowest rates were observed in Al-Baha (74.6%), Tabuk (82.8%), and Jeddah (89.8%). Notably, no health cluster achieved the national KPI 5 target of 98.0% during the study period, and three clusters recorded rates below 90.0% (Figure 3).

Because infection syndromes were not mutually exclusive, a single case may have presented with more than one concurrent diagnosis; N values for individual syndromes reflect all encounters involving that syndrome rather than unique patient counts. Notwithstanding this, acceptance rates demonstrated meaningful variation across infection syndrome categories, ranging from 82.3% to 96.5% (Table 8). The highest acceptance was observed for bloodstream and systemic infections (96.5%), central nervous system infections (95.8%), and fungal and parasitic infections (96.2%). In contrast, the lowest rates were documented for other infectious conditions (82.3%), surgical site infections (89.1%), and skin and soft tissue infections (91.4%). Statistically significant associations were identified for most categories (*p* < 0.001), except for genitourinary and sexually transmitted infections (*p* = 0.401), bone and joint infections (*p* = 0.846), and ear, eye, and throat infections (*p* = 0.925) (Table 8). Regarding indication for antibiotics, culture-guided therapy demonstrated the highest acceptance rate (96.0%), compared with empiric therapy (93.0%) and surgical prophylaxis (82.2%) (*p* < 0.001). Similarly, physician acceptance was significantly higher among cases receiving monotherapy than among those receiving combination therapy (93.4% vs. 90.5%; *p* < 0.001) (Table 8).

### 2.9. Secondary Non-KPI Assessments

#### 2.9.1. Appropriate Therapy Management

Appropriate antimicrobial therapy management was documented in 68.3% of cases with available data (n = 77,588), with statistically significant variation identified across all examined dimensions (Table 9). Temporal trends demonstrated a peak in appropriateness in 2022 (71.0%), followed by a progressive decline through 2023 (68.7%) and 2024 (65.4%), reverting toward the 2021 baseline of 65.0% (*p* < 0.001).

Institutional factors were significantly associated with documented therapy management status (*p* < 0.001). Category C hospitals recorded the highest proportion of cases classified as appropriately managed (74.2%), followed by category B (68.6%) and category A (62.3%). However, these unadjusted differences should be interpreted cautiously. Because therapy management status was based on ASP team documentation at the time of review, lower apparent appropriateness in category A hospitals may reflect greater patient complexity and/or more intensive detection and documentation of optimization opportunities by more mature stewardship teams, rather than poorer stewardship performance. Substantial geographic variation was evident, with appropriateness ranging from 35.4% in Taif to 87.3% in Al-Jouf (Table 9). Non-critical care wards demonstrated higher appropriateness than critical care units (72.2% vs. 63.2%; *p* < 0.001). Infection syndrome was significantly associated with appropriateness across most diagnostic categories (Table 9). The highest rates were observed for ear, eye, and throat infections (77.8%) and pneumonia and respiratory infections (72.2%), while the lowest were documented for surgical site infections (57.9%), other infectious conditions (60.8%), and central nervous system infections (62.2%). Bloodstream and systemic infections did not demonstrate a statistically significant difference (*p* = 0.206). Culture-guided therapy was associated with the highest appropriateness (71.9%), followed by empiric therapy (67.6%) and surgical prophylaxis (62.2%) (*p* < 0.001). Monotherapy was associated with higher appropriateness than combination therapy (69.4% vs. 62.4%; *p* < 0.001) (Table 9).

#### 2.9.2. Therapeutic Drug Monitoring Utilization and Its Impact on Antimicrobial Dose Adjustment

**Figure 4:**
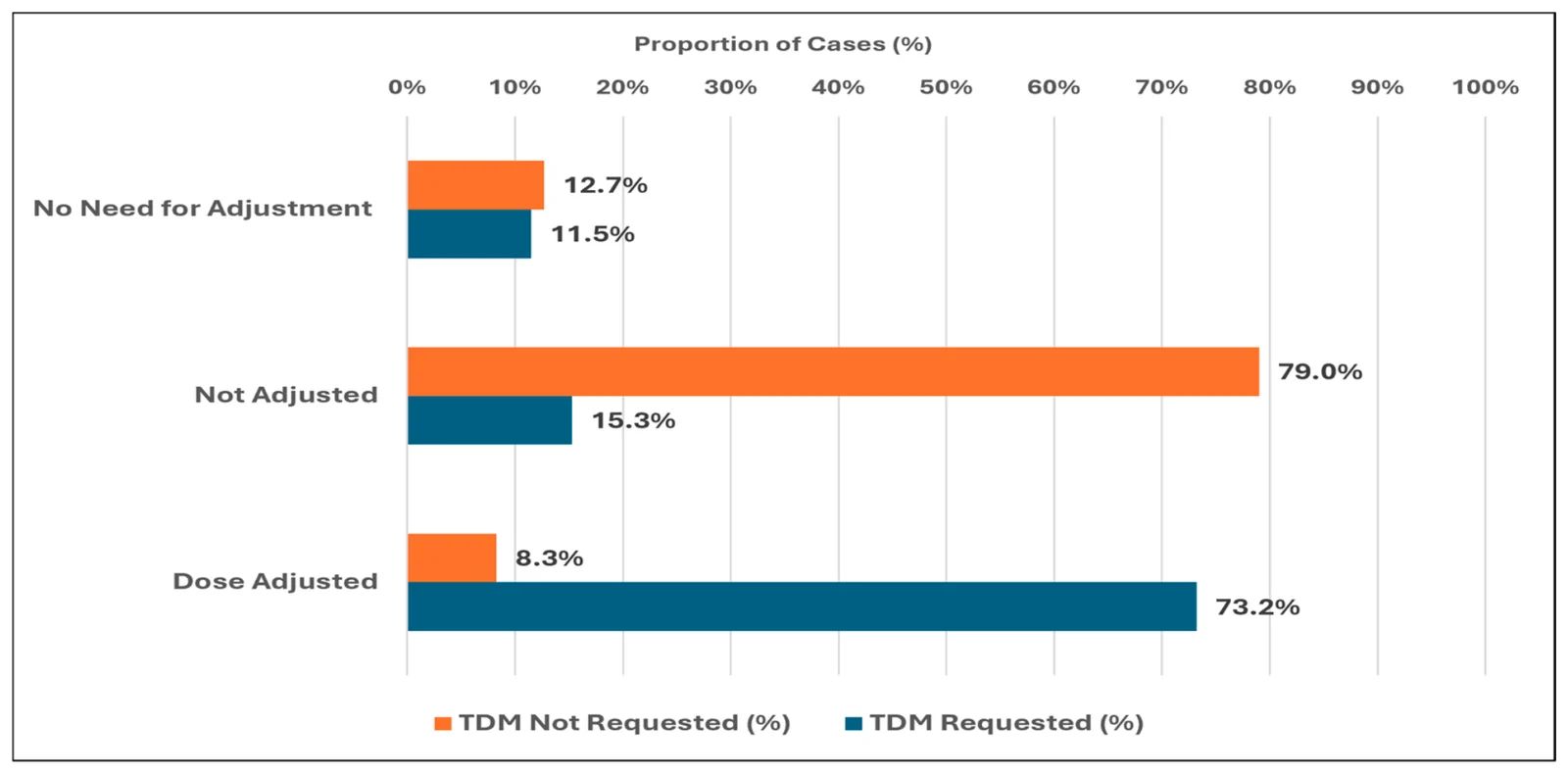
Dose-adjustment outcomes according to tdm request status among vancomycin and aminoglycoside recipients, Saudi MOH hospitals, 2021–2024. Analysis restricted to 18,092 cases with complete data for both variables, comprising cases where TDM was requested (n = 8272), TDM was not requested (n = 9565), and TDM was not required (n = 255). Figure displays findings for the TDM-requested and TDM-not-requested groups only, as the TDM-not-required group (n = 255) represents cases where systemic monitoring was clinically unnecessary. Dark teal bars represent cases where TDM was requested (n = 8272); orange bars represent cases where TDM was not requested (n = 9565). Dose adjusted: appropriate dose modification implemented following antibiotic level result; not adjusted: dose was not modified despite available level; no need for adjustment: antibiotic level within therapeutic range. The 2105 cases excluded from this analysis had missing data for dose-adjustment outcome. Chi-square *p* < 0.001.

Therapeutic drug monitoring is generally indicated for patients receiving systemic vancomycin or aminoglycosides, except for locally administered formulations or other clinical circumstances where systemic monitoring is not required. Of the 20,197 eligible cases, TDM was requested in 44.5% (n = 8990), not requested in 53.7% (n = 10,841), and was not required in 1.8% (n = 366) (Table 3). The relationship between TDM request status and subsequent dose adjustment was examined among 18,092 cases with complete data available for both variables, comprising cases where TDM was requested (n = 8272), not requested (n = 9565), and not required (n = 255). A statistically significant association was identified (*p* < 0.001). Among the 8272 cases in which TDM was requested, 73.2% (n = 6057) had the dose adjusted appropriately based on the antibiotic level, 15.3% (n = 1267) did not have the dose adjusted despite a result being available, and 11.5% (n = 948) did not require dose adjustment. In contrast, among cases where TDM was not requested (n = 9565), appropriate dose adjustment was documented in only 8.3% (n = 791); the vast majority (79.0%, n = 7561) received no dose modification and 12.7% (n = 1213) required no adjustment (Figure 4).

### 2.10. Predictors of Guideline Non-Adherence and Physician Non-Acceptance of ASP Recommendations

**Figure 5:**
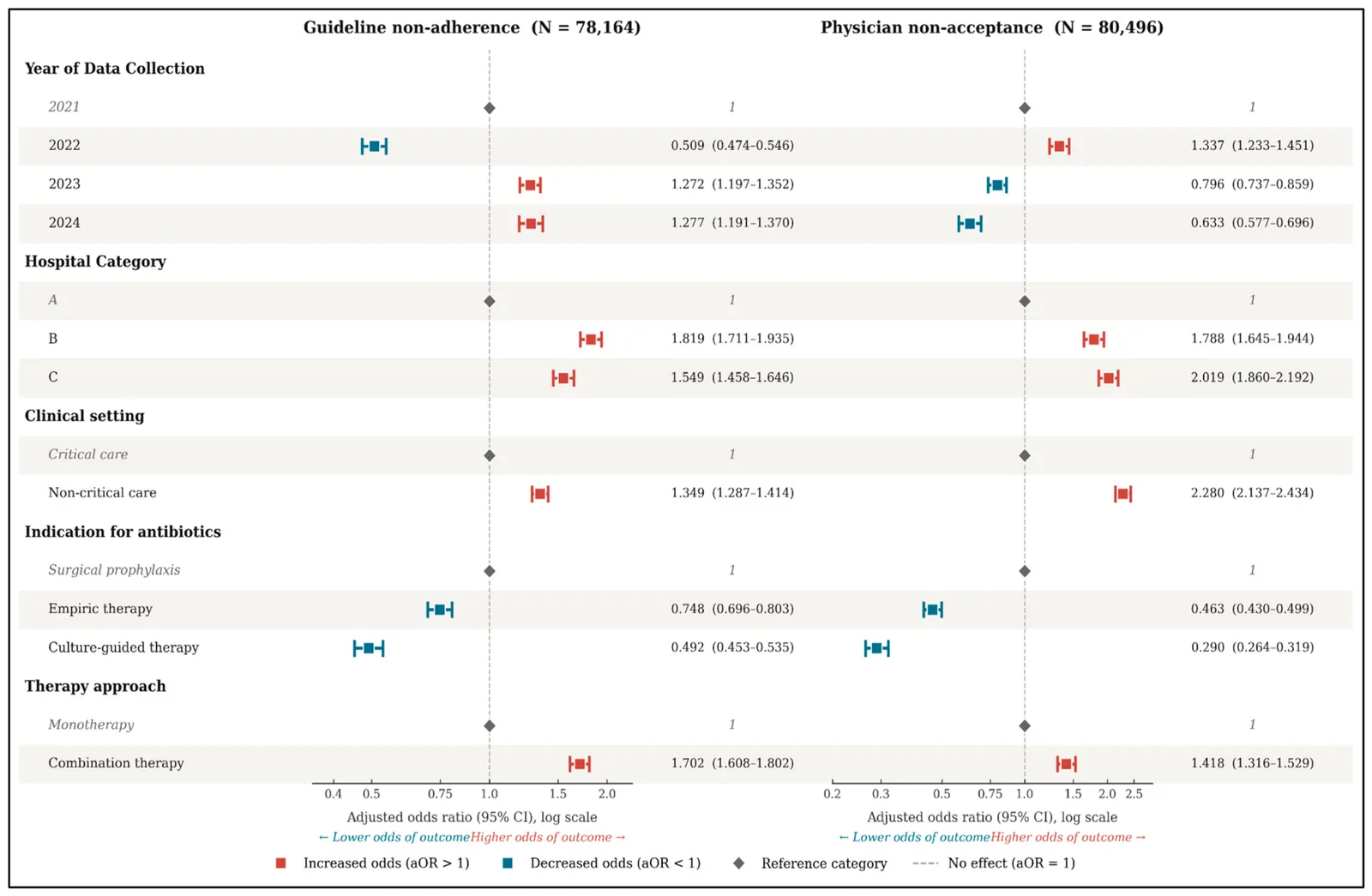
Forest plot of multivariable binary logistic regression analyses identifying independent predictors of guideline non-adherence (N = 78,164; left panel) and physician non-acceptance of ASP recommendations (N = 80,496; right panel), Saudi MOH hospitals, 2021–2024. Squares represent adjusted odds ratios (aORs) and horizontal lines depict 95% confidence intervals (CIs). Red squares indicate increased odds of the outcome (aOR > 1); teal squares indicate decreased odds (aOR < 1). Diamonds denote reference categories. The dashed vertical line at aOR = 1 represents no effect. Reference categories were 2021, hospital category A, critical care ward, surgical prophylaxis, and monotherapy. All point estimates with 95% confidence intervals excluding 1 reached statistical significance (*p* < 0.001). ASP, antimicrobial stewardship programme.

Two multivariable binary logistic regression models were performed to identify independent predictors of physician non-acceptance of ASP recommendations and guideline non-adherence (Table 10, Figure 5). For physician non-acceptance, 80,496 of 84,747 eligible cases with active ASP recommendations were included, while 4251 (5.0%) were excluded because of missing data. The model was statistically significant overall (omnibus χ^2^ = 2801.150, df = 9, *p* < 0.001), with modest explanatory power (Cox and Snell R^2^ = 0.034; Nagelkerke R^2^ = 0.086). After adjustment, year of data collection, hospital category, clinical setting, indication for antibiotics, and therapy approach all remained significantly associated with physician non-acceptance. Non-acceptance was more likely in 2022, in category B and C hospitals, in non-critical care wards, and with combination therapy, but less likely in 2023 and 2024 and in empiric and culture-guided therapy relative to surgical prophylaxis.

For guideline non-adherence, 78,164 of 81,995 eligible prescriptions were included, while 3831 (4.7%) were excluded because of missing data. This model was also statistically significant (omnibus χ^2^ = 2425.897, df = 9, *p* < 0.001), with modest explanatory power (Cox and Snell R^2^ = 0.031; Nagelkerke R^2^ = 0.058). After adjustment, year of data collection, hospital category, clinical setting, indication for antibiotics, and therapy approach all remained significantly associated with guideline non-adherence. Non-adherence was more likely in 2023 and 2024, in category B and C hospitals, in non-critical care wards, and with combination therapy, whereas lower odds were observed in 2022 and in empiric and culture-guided therapy relative to surgical prophylaxis. Overall, lower ASP capacity, non-critical care wards, and combination therapy were consistently associated with poorer stewardship-related outcomes across both models.

### 2.11. Hospital-Level Antimicrobial Use

**Figure 6:**
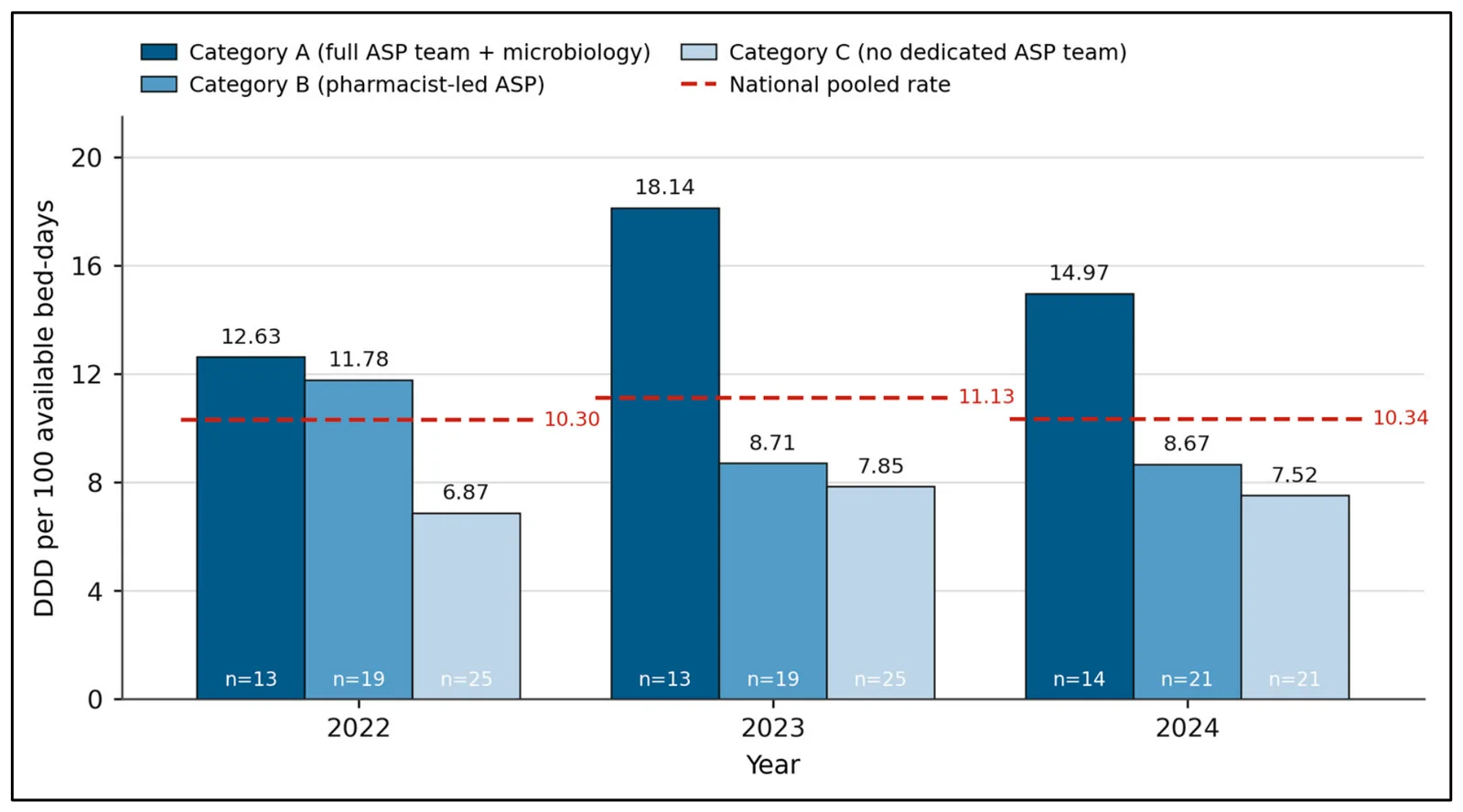
Hospital-level use of monitored Watch and reserve antimicrobial agents, expressed as defined daily doses per 100 available bed-days, by year and hospital ASP capacity category, Saudi Ministry of Health hospitals, 2022–2024. Bars show the pooled rate for each category; the number of reporting hospital units is given within each bar. The dashed line indicates the national pooled rate for that year. Category A, full ASP team with microbiology laboratory; category B, partial ASP core-team capacity; category C, no dedicated ASP team.

Antimicrobial-use returns covered the period from 2022 to 2024 for five monitored agents: meropenem, imipenem–cilastatin, piperacillin–tazobactam, vancomycin, and colistin. Use was expressed as DDD per 100 available bed-days, as defined in Section 4.6.2. The total available bed-days represented 6,635,700 in 2022, 6,635,700 in 2023, and 6,750,626 in 2024. Total use of the five monitored agents was 10.30, 11.13, and 10.34 DDD per 100 available bed-days in 2022, 2023, and 2024, respectively. The corresponding median hospital-level rates were 5.90, 7.52, and 5.98, with interquartile ranges of 3.14–13.28, 4.00–12.56, and 3.04–11.14, respectively (Table 11; Figure 6). Antimicrobial use differed significantly across ASP capacity categories in each year and was consistently highest in category A hospitals and lowest in category C hospitals. Rates for categories A, B, and C were 12.63, 11.78, and 6.87 DDD per 100 available bed-days in 2022 (*p* = 0.042); 18.14, 8.71, and 7.85 in 2023 (*p* = 0.008); and 14.97, 8.67, and 7.52 in 2024 (*p* = 0.032).

Watch-group agents accounted for 93.6–94.5% of the use of the five monitored agents across the three years. Piperacillin–tazobactam was the most frequently used monitored agent, accounting for 34.4–39.3% of total monitored use, followed by meropenem (27.7–30.0%) and vancomycin (21.5–25.2%). Colistin, the sole monitored reserve agent, accounted for 5.5–6.4% of monitored use, corresponding to 0.57–0.66 DDD per 100 available bed-days. Colistin use was highest in category A hospitals in each year, although the difference across hospital categories reached statistical significance only in 2023 (*p* = 0.032). Among the 53 hospital units reporting in all three years, median use declined from 5.97 to 5.71 DDD per 100 available bed-days, with lower use recorded in 32 of 53 units; this change was not statistically significant (*p* = 0.166). A statistically significant decline was confined to category B hospitals, in which median use fell from 6.79 to 4.84 DDD per 100 available bed-days (*p* = 0.045).

## 3. Discussion

This four-year multicentre surveillance study evaluated national ASP performance across 96 Saudi Ministry of Health hospitals, covering 124,452 patient records from 2021 to 2024. The principal finding is that stewardship performance is strongly conditioned by ASP infrastructure category. Category A hospitals demonstrated more favourable performance in protocol adherence (KPI 1), restricted-antibiotic dispensing compliance (KPI 2), physician acceptance of ASP recommendations (KPI 5), and active stewardship intervention. However, the unadjusted appropriateness measures did not follow the same gradient. Appropriateness of the initial antimicrobial regimen was highest in category C hospitals, followed by category A and category B hospitals, while appropriate ongoing therapy management was highest in category C hospitals and lowest in category A hospitals. Because these appropriateness measures were based on ASP-team documentation and were not independently validated, the observed differences may reflect variation in case complexity, documentation practices, and the intensity with which stewardship teams identified optimization opportunities, rather than true differences in prescribing quality. Multivariable adjustment for calendar year, clinical setting, antibiotic indication, and therapy approach did not eliminate this gradient: adjusted odds ratios for guideline non-adherence were 1.82 for category B and 1.55 for category C versus category A, and adjusted odds ratios for physician non-acceptance were 1.79 for category B and 2.02 for category C versus category A. These results suggest that ASP infrastructure is an important correlate of stewardship performance after adjustment for available covariates; however, residual confounding by case mix, illness severity, referral status, hospital occupancy, and local workflow cannot be excluded. Accordingly, the following discussion interprets the findings primarily according to hospital ASP capacity, while considering clinical setting, antibiotic indication, antimicrobial-use patterns, and regional variation as complementary contextual factors.

At the aggregate level, several performance indicators met their national targets, including protocol adherence (KPI 1) and the active intervention rate (KPI 3), while important gaps persisted: restricted-antibiotic dispensing did not reach the 100% target, physician acceptance did not meet the 98% target, therapeutic drug monitoring was requested in fewer than half of eligible vancomycin or aminoglycoside cases (44.5%), and stewardship optimization interventions did not show a sustained increasing trend. Taken together, these findings indicate favourable performance across several measured stewardship process indicators, although performance varied across hospital categories, regions, clinical settings, and prescribing indications. Given the large sample size of this study, statistical significance was achieved for virtually all comparisons; findings should therefore be interpreted primarily in terms of the absolute percentage differences between groups and the adjusted odds ratios with 95% confidence intervals from the multivariable analyses, rather than placing interpretive weight on *p*-values alone. Because patient-centred clinical outcomes were not assessed, these findings should not be interpreted as evidence of broader clinical effectiveness.

Overall protocol adherence exceeded the national KPI target of 80% in all study years (87.9% in 2021, 92.0% in 2022, 83.0% in 2023, and 85.4% in 2024). This suggests that ASP processes were successful in aligning prescribing with MOH or hospital guidance. The Global Point Prevalence Survey, which reported an overall guideline-compliance rate of 77.4% among adult hospital antimicrobial prescriptions across 53 countries, is in good agreement with our data [28]. However, adherence rates varied across the prescribing context. Category A hospitals, those with complete ASP teams and on-site microbiology laboratories, recorded the highest adherence (91.9%), notably exceeding both category B (85.1%) and category C (86.5%) hospitals; the comparable rates between B and C suggest that pharmacist-led ASP alone, without microbiology and physician integration, may not produce additive gains over the unstructured category C model. This pattern is consistent with IDSA/SHEA recommendations that emphasize the importance of multidisciplinary expertise, collaborative leadership between physicians and pharmacists, microbiological input, and feedback systems as fundamental components of successful stewardship programmes [25]. For surgical prophylaxis, adherence rates were lower (83.0%) compared to those for culture-guided or empiric therapy, which is concerning due to the continued contribution of surgical site infections in healthcare-related morbidity. International guidelines for surgical prophylaxis have identified inappropriate agent selection, dosing, timing issues, and antibiotic treatment duration as critical areas for further research and improvement [29]. Similarly, quantitative research demonstrates the magnitude of this issue; Lim et al. (2015) found that 66.3% of surgical prophylaxis prescriptions were inappropriate in standard clinical practice [30]. While the adherence rate in this study seems to be higher, direct numerical comparisons are constrained by variations in study design, definitions of appropriateness, and healthcare setting. Both findings, however, support surgical prophylaxis as a priority area for targeted stewardship intervention.

Additionally, protocol adherence varied by infection syndrome, with lower adherence in less well-defined categories such as other infectious conditions and greater rates in central nervous system, pneumonia/respiratory, and bloodstream/systemic infections. This pattern aligns with stewardship principles that favour syndrome-specific recommendations to standardize prescribing for common infections [25,31]. Likewise, studies have shown that adherence varies depending on the type of infection, which can be attributed to variations in the clarity of the diagnosis, the availability of guidelines, and the complexity of the prescription [32,33]. Adherence was also higher with monotherapy than with combination therapy (88.2% vs. 81.4%). This is probably because guideline recommendations emphasize targeted therapy and de-escalation to the narrowest effective regimen when clinically appropriate, as well as the additional complexity of broader empirical regimens [25]. Accordingly, lower adherence among combination regimens should be interpreted as a marker of prescribing complexity rather than as direct evidence that combination therapy was inappropriate in all cases.

Restricted-antibiotic dispensing compliance reached 87.6%, falling short of the national 100% target and showing substantial variation across hospital categories and health clusters. Higher compliance in category A hospitals than in category B hospitals suggests that the well-developed ASP infrastructure, including interdisciplinary teams and microbiology support, may improve the enforcement of restriction policies. This interpretation is consistent with data from a national survey that demonstrates inconsistent ASP implementation in Saudi hospitals, including differences in fundamental interventions such as antibiotic restriction and preauthorization procedures [27].

Regional variation was also evident across health clusters, particularly for protocol adherence and restricted-antibiotic dispensing compliance. Protocol adherence ranged from 68.1% in Hafar AlBatin to 96.0% in Al-Jouf, while restricted-antibiotic compliance ranged from 42.5% in Hafar AlBatin to 97.7% in Makkah. Such variation suggests that national ASP implementation may not be uniform across regions, even within a centralized MOH system. Differences in local ASP maturity, availability of infectious diseases and clinical pharmacy expertise, microbiology and diagnostic capacity, electronic approval workflows, documentation quality, institutional leadership, and prescriber engagement may all contribute to inter-cluster performance differences. The most pronounced gaps were observed in Hafar AlBatin, where protocol adherence reached only 68.1% and restricted-antibiotic compliance only 42.5%, in contrast to Al-Jouf (96.0% adherence) and Makkah (97.7% compliance); these extremes likely reflect differences in ASP maturity, infrastructure, and available resources rather than differences in patient case mix and represent priority targets for regional capacity-building efforts. According to Charani et al. (2019), contextual determinants of stewardship practice that may impede adherence to restriction policies even after formal programme implementation include hierarchical cultures, communication difficulties, and a lack of feedback systems [34].

Clinical setting further shaped restricted-antibiotic compliance. Higher compliance in critical care may be linked to closer monitoring, interdisciplinary decision-making, and the prioritization of high-risk patients, while lower compliance in non-critical care settings may indicate less stringent prescribing control and more varied stewardship engagement. In high-risk inpatient settings where antibiotic usage is high and necessitates close monitoring, international guidelines advocate the implementation of stewardship interventions [25]. In addition, an ICU-based prospective audit-and-feedback study demonstrated that systematic stewardship activities can improve prescribing appropriateness in critical care settings [35]. Furthermore, culture-guided therapy had the highest compliance rate, demonstrating the importance of microbiological validation in strengthening tailored prescribing decisions [36]. Even though the 100% target is significant as a national benchmark, the observed diversity suggests that restriction-policy performance should be interpreted considering local infrastructure, workflow, and documented clinical outliers. In the Saudi MOH context, these variations may also be due to differences in the ASP team availability, documentation systems, and approval protocols among hospitals.

Our data showed that the rate of active interventions reached 64.6%, surpassing the national goal of 60%. This indicates that ASP teams were not only evaluating prescriptions but also actively adjusting antimicrobial therapies. The interventions most frequently carried out included therapy optimization, dose adjustments, management-plan choices, culture requests, and antibiotic discontinuation, all of which represent essential stewardship activities aimed at optimizing antimicrobial usage. This pattern corresponds with the widely accepted definition of antimicrobial stewardship as coordinated efforts focused on optimizing the selection, dosage, duration, and route of administration of antibiotics [25,37]. The prevalence of interventions related to optimization and dosing indicates that numerous prescriptions needed modifications instead of outright replacement, emphasizing the practical significance of prospective audits and feedback.

The observed decrease in active interventions in 2022 may be attributable to residual disruptions in hospital services and stewardship workflows following the COVID-19 pandemic. It also coincided with phased expansion of mandatory ASP implementation, which could have introduced transitional variability in programme performance. Comparable disruptions in stewardship activities during and after the pandemic have been documented internationally, including alterations in stewardship capacity and prescribing practices [38,39]. As the current dataset did not directly assess the causes of this decrease, this explanation should be regarded as hypothesis-generating.

The three stewardship-driven optimization interventions comprising KPI 4, de-escalation, antibiotic discontinuation, and intravenous-to-oral conversion, did not demonstrate a sustained increasing annual trend. This contrasts with evidence that de-escalation and intravenous-to-oral switch are acknowledged stewardship goals with proven advantages in hospital settings, albeit the degree of certainty differs depending on the intervention [21,23]. At the same time, the progressive decline in antibiotic discontinuation and the late drop in de-escalation in 2024 are consistent with data showing that recommendations for lowering antimicrobial exposure are frequently more difficult to implement than those pertaining to continuation or escalation. Langford et al. (2020) found that ASP recommendations aimed at decreasing antibiotic exposure were less likely to be accepted [40]. Similarly, Shively et al. (2022) observed reduced acceptance rates for recommendations to discontinue or de-escalate antimicrobials [41]. Therefore, focused methods that increase acceptability of exposure-reducing interventions, backed by prompt microbiological evaluation and organized audit-and-feedback procedures, may be necessary for sustained improvement in KPI 4.

The national KPI 5 target of >98% physician acceptance is ambitious relative to published benchmarks. The observed rate of 93.0%, although below the national target, compares favourably with the international ASP literature. Langford et al. (2020) reported an overall acceptance rate of 82.2% in a large prospective audit and feedback programme, with acceptance varying substantially by clinical service and recommendation type, while Shively et al. (2022) reported a TeleASP recommendation acceptance rate of 91.2% overall, ranging from 86.4% in general surgery to 97.5% in critical care [40,41]. In this context, the 93.0% acceptance observed across 96 hospitals and 17 health clusters reflects strong physician engagement with ASP recommendations. The remaining gap relative to the 98% benchmark reflects the challenge of achieving near-universal acceptance across diverse clinical settings, infection types, and recommendation categories rather than evidence of poor physician cooperation.

Critical care wards had a higher acceptance rate (96.2% vs. 89.9%) than non-critical care wards. Given that critical care settings frequently rely on interdisciplinary decision-making, frequent reassessment, and real-time feedback, this is clinically plausible. Over a ten-year period, Sehgal et al. (2021) [42] showed that antimicrobial stewardship recommendations were consistently accepted in intensive care, and Shively et al. (2022) observed a particularly high level of acceptability among critical care medicine prescribers [41].

Physician acceptance also differed by infection syndrome, with lower acceptance in other infectious conditions, surgical site infections, and skin/soft tissue infections, and higher acceptance in bloodstream/systemic, CNS, and fungal/parasitic infections. This result should be interpreted descriptively because there is currently little direct comparison data connecting infection syndrome to acceptance of ASP recommendations. Our finding that category A hospitals had the greatest acceptance rate (96.4%) further supports the importance of a well-established ASP framework. According to IDSA/SHEA guidelines, ASPs are ideally led by infectious diseases specialists along with pharmacist colleagues with stewardship competencies to ensure smooth cooperation and compliance to recommendations [25]. The lower rate of acceptance among cases receiving combination therapy as opposed to monotherapy (90.5% vs. 93.4%) may reflect greater clinical complexity and prescriber caution in patients requiring broader antimicrobial coverage. This interpretation is consistent with Langford et al. (2020), who found lower acceptance of recommendations to decrease antibiotic exposure than of recommendations to increase exposure [40].

As a secondary non-KPI assessment, appropriate antimicrobial therapy management was documented in 68.3% of cases with available data, while nearly one-third were classified as requiring stewardship optimization. This finding complements the active-intervention results by showing that stewardship evaluation extends beyond whether interventions are made to whether ongoing therapy is adjusted, monitored, escalated, de-escalated, discontinued, or converted from intravenous-to-oral therapy when clinically indicated. However, this measure should be interpreted cautiously because it was based on the ASP team documentation and was not independently verified. The lower proportion of cases classified as appropriately managed in category A hospitals may reflect greater patient complexity and referral-level infections, but it may also indicate more intensive stewardship review, whereby mature ASP teams identify and document more optimization opportunities. Conversely, higher apparent appropriateness in lower-capacity hospitals may partly reflect under-detection or under-documentation of therapy management problems. The analysis was not adjusted for illness severity, case mix, or microbiological complexity; therefore, this finding should be interpreted as a marker of intervention intensity and documentation sensitivity rather than as evidence of poorer performance in higher-capacity hospitals or superior performance in lower-capacity hospitals.

TDM represented an important stewardship gap. Among reviewed vancomycin or aminoglycoside cases, TDM was requested in only 44.5%, and more than half had no documented TDM request. Among cases with complete dose-adjustment data, appropriate dose adjustment was substantially more frequent when TDM was requested than when it was not requested (73.2% vs. 8.3%). This is significant from a clinical standpoint because current consensus guidelines recommend AUC-guided dosing and monitoring for serious MRSA infections, and international vancomycin monitoring guidelines emphasize serum concentration monitoring to maximize efficacy and minimize toxicity [43]. While excessive vancomycin exposure has been linked to an increased risk of acute kidney injury (AKI) (significantly higher AKI risk for trough concentrations ≥20 μg/mL compared to those at 15–20 μg/mL) [44], systematic-review evidence further supports the clinical usefulness of vancomycin TDM, demonstrating enhanced effectiveness and lower nephrotoxicity with monitoring [45]. The requirement for enhanced TDM processes within Gulf hospitals is additionally underscored by regional information from Oman, which indicated that the inability to reach targeted vancomycin blood concentrations was frequent and associated with worse outcomes [46].

Quantifying hospital-level antimicrobial use allowed stewardship process performance to be considered alongside use of the agents that ASPs are intended to protect. Capacity-adjusted use of the five monitored agents remained broadly stable at approximately 10–11 DDD per 100 available bed-days from 2022 to 2024. Among the 53 hospital units reporting in all three years, the overall decline was small and not statistically significant, while a statistically significant reduction was observed only among category B hospitals. Use of the monitored agents, including colistin, was consistently highest in category A hospitals and lowest in category C hospitals. Category A hospitals had greater bed capacity and may have managed patients with more complex infections or different referral patterns. However, case mix, illness severity, referral status, and hospital occupancy were not available for adjustment. Higher use in category A hospitals therefore cannot be attributed definitively to greater clinical complexity or interpreted as evidence of poorer stewardship performance. These findings support targeted surveillance of broad-spectrum and reserve-agent use in higher-capacity hospitals, ideally using occupancy-adjusted denominators and case-mix information [47].

Use was concentrated in piperacillin–tazobactam and meropenem, which together accounted for approximately two-thirds of the use of the five monitored agents in each year. These agents therefore represent important targets for prospective audit, review of treatment duration, and de-escalation when microbiological and clinical findings permit. However, intravenous-to-oral conversion is not generally applicable directly to meropenem or piperacillin–tazobactam and should not be presented as a specific optimization strategy for these agents. Comparisons with published hospital-use estimates should also be made cautiously because the present analysis used available rather than occupied bed-days and captured only five selected agents.

Finally, multivariable regression analyses identified independent predictors of guideline non-adherence and physician non-acceptance of ASP recommendations. These models supported several patterns observed in the descriptive KPI analyses: lower ASP capacity, non-critical care setting, and combination therapy were consistently associated with poorer stewardship-related outcomes. The modest explanatory power of both models indicates that unmeasured factors, including illness severity, local workflow, prescriber specialty, staffing, and institutional culture, likely influenced stewardship performance. This aligns with qualitative evidence highlighting the role of organizational context in ASP implementation [34].

Category A hospitals demonstrated consistently superior performance across multiple stewardship indicators, including protocol adherence (91.9% vs. 85.1% and 86.5% in categories B and C), restricted-antibiotic dispensing compliance (90.7% vs. 85.0%), and physician acceptance of ASP recommendations (96.4% vs. 92.3% and 91.2%). Multivariable analysis confirmed that category B and C hospitals had significantly higher adjusted odds of guideline non-adherence (aOR 1.82 and 1.55 respectively) and physician non-acceptance (aOR 1.79 and 2.02, respectively) compared with category A, even after adjusting for year, clinical setting, indication, and therapy approach. This consistent pattern supports targeted national investment in lower-capacity hospitals, including expansion of infectious diseases expertise, stewardship pharmacy capacity, microbiology laboratory support, and standardized electronic approval workflows. These findings should be interpreted as infrastructure-associated differences in stewardship process performance rather than as evidence of superior patient-level clinical outcomes in category A hospitals.

Overall, the findings of this study indicate that the national ASP achieved meaningful progress in measured stewardship performance indicators, including protocol adherence, intervention activity, and physician engagement. At the same time, persistent gaps in restricted-antibiotic dispensing, exposure-reducing interventions, TDM implementation, and inter-hospital variability still exist. Because patient-level outcomes were not available, the next phase of national ASP evaluation should link stewardship performance indicators with clinical outcomes, including mortality, length of stay, readmission, adverse drug events, *Clostridioides difficile* infection, nephrotoxicity, and infection-related outcomes.

### Strengths and Limitations

To our knowledge, this is one of the largest nationwide evaluations of antimicrobial stewardship in the Gulf region and the first to assess the Saudi national programme against all five Ministry of Health key performance indicators simultaneously. Using 124,452 patient records collected over four years across 17 health clusters, the study provides a broad national surveillance overview of MOH hospitals actively reporting ASP activities. However, non-reporting or inconsistently reporting hospitals were not captured. Stratifying hospitals by ASP infrastructure, categories A, B, and C, enabled assessment of how stewardship capacity relates to prescribing quality, intervention activity, and physician engagement, which are directly relevant to national workforce planning. Evaluating all five KPIs alongside secondary measures of therapy management, therapeutic drug monitoring, and antimicrobial use provided a more comprehensive picture than single-indicator studies. The multivariable models also added insight into where stewardship efforts may need to be intensified.

The study has several limitations. Because the data were collected routinely for surveillance rather than research, documentation varied between hospitals and some variables had missing values. For example, hospital category was missing for 4.2% of records, and several outcomes were analyzed on an available-case basis. The study could not independently verify whether each recorded diagnosis represented a confirmed bacterial infection. The national ASP surveillance dataset did not capture inflammatory biomarkers, diagnostic imaging, viral testing results, or the specific clinical criteria used to initiate empiric therapy. Assessments of guideline adherence and regimen appropriateness therefore reflect the ASP team’s documented judgement at the time of pharmacist review and were not independently validated against full clinical records. Future evaluations linking ASP surveillance data with electronic health records and microbiological datasets would allow more rigorous assessment of the appropriateness of antibiotic initiation.

The dataset also did not include culture-specific information, such as specimen counts, culture positivity rates, organism identification, or susceptibility results. Although the proportion of cases classified as culture-guided therapy, 25.7%, provides a broad indication of microbiological input, it was not possible to evaluate the quality or appropriateness of culture-guided decisions at the individual-patient level. In addition, the study may be subject to selection bias because it included MOH hospitals that were actively reporting ASP activities during the study period. Hospitals with weaker stewardship infrastructure, incomplete documentation systems, limited personnel, or inconsistent reporting may have been underrepresented or excluded. Consequently, the findings may overestimate national ASP performance if reporting hospitals had more mature ASP implementation or stronger administrative capacity than non-reporting hospitals. The results should therefore be interpreted as reflecting ASP performance among reporting MOH hospitals, rather than all MOH hospitals or all Saudi healthcare facilities.

The large sample size, N = 124,452, means that even clinically trivial differences between groups may produce *p*-values below conventional significance thresholds. Statistical significance in the descriptive cross-tabulations, Table 4, Table 5, Table 6, Table 7, Table 8 and Table 9, should therefore not be equated with clinical or operational meaningfulness. Absolute percentage differences and the adjusted odds ratios with 95% confidence intervals reported in the multivariable logistic regression models provide more informative measures of effect size and should form the main basis for interpreting the practical significance of observed associations.

The primary analytical unit was the patient prescription, consistent with the national KPI framework. However, prescriptions are nested within hospitals, and hospitals within health clusters; therefore, the assumption of independent observations required for standard chi-square tests and binary logistic regression may not be fully met. Intra-cluster correlation may have underestimated standard errors and inflated the apparent statistical precision of some estimates. Future national ASP evaluations should consider multi-level modelling approaches that explicitly account for this hierarchical data structure, or supplement patient-level analyses with hospital-level performance summaries presented as median and interquartile range across participating hospitals.

The interpretation of KPI 1 is also limited by the exclusion of prescriptions classified as outside available protocol indications, which accounted for 34.1% of all reviewed prescriptions. This exclusion was methodologically necessary to avoid classifying prescriptions without an applicable protocol as adherent or non-adherent. However, it may have increased the reported protocol-adherence estimate among eligible prescriptions. KPI 1 should therefore be interpreted as protocol adherence among protocol-covered diagnoses, rather than as a measure of protocol coverage or overall appropriateness across all antimicrobial use. The large proportion of prescriptions outside protocol indications (34.1%), predominantly comprising bloodstream and systemic infections without a dedicated protocol, highlights a meaningful gap in national protocol coverage and supports future expansion of syndrome-specific antimicrobial guidance to include sepsis and bacteraemia management.

The low frequency of documented resistance indicators should be interpreted cautiously. These values do not represent isolate-level AMR prevalence and should not be interpreted as evidence of a low resistance burden in Saudi hospitals. Instead, they reflect documented resistance flags among all ASP-reviewed antibiotic-treated cases. Because cultures and susceptibility testing were not systematically performed or captured for all cases, particularly for empiric therapy and surgical prophylaxis, resistance may have been under-ascertained. Future ASP evaluations should be linked with isolate-level microbiology datasets to allow pathogen-specific, specimen-specific, and antimicrobial-specific resistance reporting using standardized AMR surveillance denominators.

The hospital-level antimicrobial-use analysis also has several limitations. First, the returns covered only five monitored broad-spectrum agents and did not include Access-group antibiotics; therefore, the findings do not represent total hospital antimicrobial use or permit full assessment of the national AWaRe distribution. Second, antimicrobial-use returns were available from 60 separately reporting hospital units representing 58 of the 96 participating hospitals and covered only 2022–2024, preventing direct alignment with the 2021 stewardship data. Third, occupied bed-days and admissions, which are preferred denominators for standardized hospital antimicrobial-use surveillance, were unavailable [48]. Use was therefore expressed as DDD per 100 available bed-days based on licenced capacity. Because available bed-days exceed occupied bed-days when occupancy is below 100%, the reported rates are likely to underestimate rates based on occupied bed-days, and differences in occupancy across hospitals and years could not be assessed. Two implausible agent-level values were excluded because they could not be verified against the original returns. Because the data were aggregated at the hospital-unit level, antimicrobial use could not be linked to individual prescriptions, clinical indications, illness severity, case complexity, or patient-level outcomes. Future national surveillance should capture occupied bed-days and admissions, extend reporting to Access-group agents, and link antimicrobial-use returns with prescription-level ASP and microbiological data.

Finally, although the regression models reached statistical significance, they explained only a modest proportion of the variance, with Nagelkerke R^2^ values of 0.06–0.09. This indicates that unmeasured factors, such as illness severity, prescriber specialty, staffing, and local culture, may also shape stewardship performance. The dataset did not include patient-level outcomes such as length of stay, mortality, readmission, adverse drug events, vancomycin-related nephrotoxicity, *Clostridioides difficile* infection, or resistance trends. Therefore, the study cannot determine whether observed improvements in stewardship process indicators translated into improved clinical outcomes. The findings should consequently be interpreted as evidence of ASP process performance and descriptive antimicrobial-use patterns, rather than evidence of patient-level clinical effectiveness.

## 4. Materials and Methods

### 4.1. Study Design

This retrospective, observational, multicentre surveillance analysis evaluated national ASP performance indicators and secondary stewardship assessments across Saudi MOH hospitals from 2021 to 2024.

### 4.2. Study Setting

The study was conducted across 96 Saudi MOH hospitals that were actively reporting ASP activities to the MOH during the study period from 2021 to 2024. Therefore, the study population represents hospitals participating in the national ASP surveillance and reporting system, rather than all MOH hospitals in Saudi Arabia. Participating hospitals were distributed across the five geographic regions of Saudi Arabia: Central, Eastern, Western, Northern, and Southern regions.

Hospital characteristics included:

- Bed capacity ranging from 50 to 800 beds, with an average capacity of 300 beds.
- Hospital type: 44 general hospitals (45.8%), 33 central hospitals (34.4%), 16 specialized hospitals (16.7%, including pediatric, maternity, and cardiac centres), and 3 referral hospitals (3.1%).
- Hospitals categorized according to the availability of ASP core elements and resources:
  ▪ Category A: 20 hospitals (20.8%) with full ASP implementation, including a complete core team (infectious diseases physician/consultant and specialized clinical pharmacist in infectious diseases), together with the required programme components, diagnostic tools, and laboratory support.
  ▪ Category B: 26 hospitals (27.1%) with partial ASP core-team availability, where either an infectious diseases physician or a clinical pharmacist was present, together with essential diagnostic and laboratory resources. In these hospitals, ASP implementation primarily used a preauthorization strategy for restricted antibiotics.
  ▪ Category C: 50 hospitals (52.1%) with limited ASP resources and no dedicated ASP team, where only a trained pharmacist was available. The programme in these settings focused on monitoring adherence to clinical guidelines/protocols and documenting interventions related to compliance and dose optimization.
- The 17 Ministry of Health administrative health clusters were nested under the five official geographic regions of Saudi Arabia as defined by the General Authority for Statistics for reporting purposes. The Central region comprises the First, Second, and Third Health Clusters of the Central Region (Riyadh) and Al-Qassim; the Eastern region comprises the Eastern Province, Al-Ahsa, and Hafar AlBatin; the Western region comprises Makkah, Madinah, Jeddah, and Taif; the Northern region comprises Hail, Tabuk, Al-Jouf, and Northern Borders; and the Southern region comprises Aseer, Najran, Jazan, and Al-Baha. This nested presentation preserves cluster-level granularity for national programme cross-referencing while providing a regional umbrella to support international interpretation.

### 4.3. Target Population

The target population comprised all hospitalized patients who received at least one systemic antibiotic during the study period across the participating hospitals. Physicians prescribing antibiotics were also considered for analyses of acceptance of ASP recommendations.

#### 4.3.1. Inclusion Criteria

- Hospitalized patients of any age or sex.
- Receipt of at least one systemic antibiotic dose during the study period.
- Admission to critical or non-critical inpatient settings, including general medical, surgical, intensive care, or neonatal wards.

#### 4.3.2. Exclusion Criteria

- Outpatients.
- Patients receiving only topical antibiotics.
- Medical records with more than 20% missing key variables.

### 4.4. Data Source and Collection

Data were extracted retrospectively from the General Department of Pharmaceutical Care at the Saudi MOH through the national ASP surveillance dataset, which incorporated information from hospital electronic medical records, pharmacy dispensing databases, and microbiology laboratory information systems. A standardized data extraction form was used.

Hospital-level antimicrobial use data were obtained separately from national returns submitted to the General Department of Pharmaceutical Care: annually for 2022, quarterly with an annual total for 2023, and in two six-month periods for 2024. Returns report total quantity dispensed, expressed as defined daily doses, for five monitored broad-spectrum agents, and were linked to the ASP hospital master file by hospital name and health cluster to obtain licenced bed capacity. Antimicrobial-use returns covered 60 separately reporting hospital units representing 58 of the 96 participating hospitals (60.4%). Of these units, 57 reported in 2022, 57 in 2023, and 56 in 2024; 53 provided data in all three years.

### 4.5. Data Collected

The following variables were extracted:

- Demographic and institutional variables: Year of data collection, hospital category, health cluster, age group, and sex.
- Clinical and prescribing variables: Clinical setting, infection syndrome(s), antibiotic indication (empiric therapy, culture-guided therapy, or surgical prophylaxis), and therapy approach (monotherapy or combination therapy).
- Laboratory-confirmed antimicrobial resistance indicators: Multidrug-resistant organisms (MDRs), extended-spectrum beta-lactamase (ESBL), carbapenem-resistant Enterobacterales (CRE), OXA-48, New Delhi metallo-beta-lactamase (NDM), vancomycin-resistant *Enterococcus* (VRE), and extensively drug-resistant organisms (XDR). Laboratory-confirmed antimicrobial resistance indicators were extracted as binary case-level variables. For descriptive reporting, each resistance indicator was calculated as the number of ASP-reviewed antibiotic-treated inpatient cases with a documented resistance indicator divided by the total number of ASP-reviewed cases. These indicators were not calculated using isolate-level denominators, culture-positive cases, tested organisms, or organism-specific antimicrobial susceptibility-testing denominators. Therefore, they should be interpreted as documented resistance flags within the ASP surveillance dataset rather than as isolate-level AMR prevalence estimates.
- ASP evaluation variables: Guideline/protocol adherence status, appropriateness of the initial antimicrobial regimen, appropriateness of ongoing therapy management, intervention type(s), and physician response to ASP recommendations.
- Therapeutic drug monitoring variables for vancomycin and aminoglycosides: TDM request status and dose-adjustment outcome.
- Hospital-level antimicrobial use variables: The total quantity of each monitored agent dispensed, expressed in defined daily doses (DDDs) according to the WHO Anatomical Therapeutic Chemical (ATC)/DDD methodology [49]; the reporting period; and the licenced bed capacity of the reporting hospital. Monitored agents were meropenem, imipenem–cilastatin, piperacillin–tazobactam, vancomycin and colistin. Agents were grouped according to the WHO AWaRe classification [47], under which meropenem, imipenem–cilastatin, piperacillin–tazobactam and vancomycin are Watch agents and colistin is a reserve agent.

### 4.6. Measured Outcomes

#### 4.6.1. Key Performance Indicator (KPI) Assessment

Five KPIs were defined in alignment with national ASP benchmarks established by the Saudi MOH and operationalized using variables captured within the standardized ASP surveillance dataset.

- KPI 1: Protocol Adherence Rate (Target: >80%). KPI 1 was calculated only among protocol-eligible prescriptions, defined as prescriptions for diagnoses or indications that are consistent with MOH or institutional treatment guidelines/protocols at the time of ASP review. Prescriptions classified as “diagnosis outside protocol indications” were excluded from both the numerator and denominator because no applicable protocol was available against which adherence could be assessed. Therefore, KPI 1 reflects protocol adherence among protocol-covered prescriptions rather than adherence across all reviewed antimicrobial prescriptions.
- KPI 2: Restricted Antibiotic Dispensing Compliance (Target: 100%). Compliance was assessed exclusively among category A and B hospitals and was defined as the proportion of restricted-antibiotic prescriptions dispensed following documented ASP approval. Prescriptions for non-restricted antibiotics were excluded from this analysis.
- KPI 3: ASP Active Intervention Rate (Target: >60%). The active intervention rate was defined as the proportion of reviewed cases with documented intervention status that received at least one active stewardship intervention. Cases in which the regimen was reviewed and continued without modification were excluded from the numerator and were tracked separately.
- KPI 4: Stewardship-Driven Optimization Interventions (Target: Sustained Increasing Annual Trend). KPI 4 comprised three optimization interventions: de-escalation of therapy, antibiotic discontinuation, and intravenous-to-oral route conversion. Individual rates were calculated annually to assess temporal trends. A binary composite variable was additionally derived to represent the combined KPI 4 rate, defined as the presence of at least one qualifying intervention per case, thereby eliminating double-counting where multiple interventions were applied simultaneously.
- KPI 5: Physician Acceptance Rate (Target: >98%). Physician acceptance was calculated as the proportion of ASP-recommended interventions accepted by the treating physician. Cases documented as requiring no intervention were excluded from both the numerator and denominator to ensure the rate reflected genuine acceptance behaviour.

#### 4.6.2. Secondary Non-KPI Assessments

- Appropriate antimicrobial therapy management was based on the ASP team’s documented assessment at the time of review and reflected whether ongoing therapy required optimization, such as therapeutic drug monitoring, escalation, de-escalation, or intravenous-to-oral conversion. This measure was not independently reviewed and may therefore be influenced by stewardship team expertise, case complexity, documentation practices, and the intensity of ASP review.
- Therapeutic drug monitoring utilization and dose-adjustment outcomes among reviewed vancomycin or aminoglycoside cases. TDM request status was categorized as requested, not requested, or not required. Dose-adjustment outcomes were assessed among cases with complete data for both variables and categorized as dose adjusted, not adjusted, or no need for adjustment.
- Exploratory multivariable analyses to identify independent predictors of guideline non-adherence and physician non-acceptance of ASP recommendations.
- Hospital-level antimicrobial use: Antimicrobial-use returns were available for five monitored agents. Meropenem, imipenem–cilastatin, piperacillin–tazobactam, and vancomycin were classified in the World Health Organization Access, Watch and reserve (AWaRe) Watch group, while colistin was classified in the reserve group. Because occupied bed-days and admission data were unavailable, antimicrobial use was expressed as defined daily doses (DDDs) per 100 available bed-days. Available bed-days were calculated for each reporting unit as licenced bed capacity multiplied by the number of days covered by the reporting period. The pooled rate was calculated as total DDD divided by total available bed-days and multiplied by 100. Partial-year returns contributed only the days covered by the relevant reporting period, while non-reporting units were excluded from both the numerator and denominator rather than treated as having zero use. Results were reported collectively for the five monitored agents, separately by agent, and by AWaRe category. This indicator represents a capacity-adjusted hospital-level measure rather than the conventional DDD per 100 occupied bed-days. Because available bed-days exceed occupied bed-days when occupancy is below 100%, the calculated rates are expected to underestimate rates based on occupied bed-days.

### 4.7. Sample Size and Sampling Approach

Because this study used retrospective national surveillance data, no a priori sample-size calculation was performed. A census approach was adopted in which all eligible patient records available during the study period were included after application of the prespecified exclusion criteria. This approach was intended to provide comprehensive coverage of the study population within participating MOH hospitals where ASPs had been implemented.

### 4.8. Statistical Analysis

All statistical analyses were performed using IBM SPSS Statistics for Windows, version 20.0 (IBM Corp., Armonk, NY, USA). Categorical variables were summarized as frequencies and percentages of valid observations, with missing data excluded per variable. Bivariate associations were assessed using Pearson’s chi-square test, applied across all KPI outcomes and stratifying variables (year, hospital category, health cluster, clinical setting, infection syndrome, antibiotic indication, therapy approach). Two multivariable logistic regression models, with a priori covariates entered simultaneously, identified independent predictors of guideline non-adherence and physician non-acceptance. Fit was assessed via the omnibus chi-square test and explanatory power via Cox and Snell and Nagelkerke R^2^; results are reported as adjusted odds ratios (95% CI). Significance was set at two-sided *p* < 0.05 throughout. Hospital-level antimicrobial use was summarized as the pooled rate (total DDD ÷ total available bed-days × 100) and, given small stratum sizes and non-normal distributions, as median and interquartile range. Differences between ASP categories were assessed using the Kruskal–Wallis test, and within-hospital change from 2022 to 2024 using the Wilcoxon signed-rank test.

## 5. Conclusions

This retrospective multicentre surveillance analysis indicates that Saudi Ministry of Health hospitals actively reporting antimicrobial stewardship activities achieved favourable performance in protocol adherence, active stewardship intervention, and physician acceptance of recommendations. Hospital-level use of five monitored Watch and reserve agents, expressed as DDD per 100 available bed-days, provided complementary context for interpreting stewardship performance across ASP capacity categories. Use of these agents was consistently highest in category A hospitals; however, this finding could not be adjusted for hospital occupancy, case mix, illness severity, or referral status and should not be interpreted as evidence of either poorer stewardship or greater clinical complexity. Important gaps remained in restricted-antibiotic dispensing compliance, sustained implementation of optimization interventions, therapeutic drug monitoring, and consistency of performance across hospitals, regions, and clinical settings. Continued strengthening of ASP infrastructure, monitoring, and feedback mechanisms is warranted. Future studies should link stewardship process indicators and standardized antimicrobial-use measures with patient-level clinical outcomes, antimicrobial resistance patterns, and safety outcomes, while investigating barriers to therapeutic drug monitoring, surgical-prophylaxis optimization, and inter-cluster variation.

## Figures and Tables

**Table 1:** Demographic and institutional characteristics of patients included in the antimicrobial stewardship surveillance dataset (N = 124,452).

			N (%)
**Year of Data Collection**			
	2021		23,947 (19.2%)
	2022		25,730 (20.7%)
	2023		48,174 (38.7%)
	2024		26,601 (21.4%)
**Hospital Category**			
	A		31,610 (26.5%)
	B		39,654 (33.3%)
	C		47,914 (40.2%)
**Health Cluster**			
	**Central Region**		30,191 (24.3%)
		Riyadh	19,838 (15.9%)
		Al-Qassim	10,353 (8.3%)
	**Eastern Region**		16,357 (13.1%)
		Eastern Province	3067 (2.5%)
		Al-Ahsa	10,588 (8.5%)
		Hafar AlBatin	2702 (2.2%)
	**Western Region**		34,585 (27.8%)
		Makkah	14,937 (12.0%)
		Madinah	4828 (3.9%)
		Jeddah	12,229 (9.8%)
		Taif	2591 (2.1%)
	**Northern Region**		18,243 (14.7%)
		Hail	6387 (5.1%)
		Tabuk	4268 (3.4%)
		Al-Jouf	5239 (4.2%)
		Northern Borders	2349 (1.9%)
	**Southern Region**		25,073 (20.1%)
		Aseer	6623 (5.3%)
		Najran	2544 (2.0%)
		Jazan	5207 (4.2%)
		Al-Baha	10,699 (8.6%)
**Age Group**			
	1–12 months		11,414 (9.2%)
	1–17 years		13,178 (10.6%)
	18–24 years		7257 (5.8%)
	25–34 years		15,341 (12.3%)
	35–44 years		15,521 (12.5%)
	45–54 years		14,332 (11.5%)
	55–64 years		15,768 (12.7%)
	≥65 years		31,641 (25.4%)
**Sex**			
	Male		69,488 (55.8%)
	Female		54,964 (44.2%)

Values are presented as N (%) of valid observations. Hospitals were categorized by antimicrobial stewardship programme (ASP) capacity: category A (full ASP team and microbiology laboratory), category B (partial ASP core-team capacity, with either infectious-disease physician or clinical pharmacist involvement), and category C (no dedicated ASP team). The 17 Ministry of Health administrative health clusters were nested under the five official geographic regions of Saudi Arabia (Central, Eastern, Western, Northern, and Southern) as defined by the General Authority for Statistics; regional subtotals sum to 124,449, and three records with missing cluster information are excluded from the regional and cluster rows. The First, Second, and Third Central Region Health Clusters (Riyadh) are reported as a single merged administrative entity, consistent with their operational reporting. ASP: Antimicrobial stewardship programme.

**Table 2:** Clinical characteristics, infection types, and antimicrobial resistance patterns among the study population.

		N (%)
**Clinical setting**		
	Ward class—non-critical care	72,959 (58.6%)
	Ward class—critical care	51,493 (41.4%)
**Infection syndrome**		
	Pneumonia and respiratory infections	45,060 (36.2%)
	Bloodstream and systemic infections	29,209 (23.5%)
	Other infectious conditions	16,956 (13.6%)
	Skin and soft tissue infections	14,817 (11.9%)
	Abdominal and gastrointestinal infections	10,906 (8.8%)
	Genitourinary and sexually transmitted infections	9831 (7.9%)
	Central nervous system infections	4830 (3.9%)
	Surgical site infection (SSI)	1880 (1.5%)
	Bone and joint infections	1391 (1.1%)
	Fungal and parasitic infections	351 (0.3%)
	Ear, eye, and throat infections	139 (0.1%)
**Documented resistance indicators**		
	Multidrug-resistant (MDR)	1117 (0.9%)
	Extended-spectrum β-lactamase (ESBL)	1326 (1.1%)
	Carbapenem-resistant Enterobacterales (CRE)	917 (0.7%)
	OXA-48	102 (0.1%)
	New Delhi metallo-β-lactamase (NDM)	107 (0.1%)
	Vancomycin-resistant *Enterococcus* (VRE)	69 (0.06%)
	Extensively drug-resistant (XDR)	517 (0.4%)
**Indication for antibiotics**		
	Empiric therapy	76,055 (61.1%)
	Culture-guided therapy	31,941 (25.7%)
	Surgical prophylaxis	16,456 (13.2%)
**Therapy approach**		
	Monotherapy (1 antibiotic)	106,453 (85.7%)
	Combination therapy (≥2 antibiotics)	17,814 (14.3%)

Values are presented as N (%) of valid observations. Infection syndromes reflect the primary recorded diagnosis at the time of antimicrobial prescribing; resistance indicators are laboratory-confirmed case-level flags documented in the ASP surveillance dataset and were calculated using all valid ASP-reviewed antibiotic-treated inpatient cases as the denominator, not culture-positive isolates or organism-specific susceptibility-tested isolates. Ward class denotes the level of care at treatment initiation. XDR, extensively drug-resistant; MDR, multidrug-resistant; ESBL, extended-spectrum beta-lactamase; CRE, carbapenem-resistant Enterobacterales; OXA-48, oxacillinase-48; VRE, vancomycin-resistant *Enterococcus*; NDM, New Delhi metallo-β-lactamase.

**Table 3:** Antimicrobial stewardship evaluation among reviewed antibiotic prescriptions, stratified by hospital ASP capacity (N = 119,178).

		Category A n (%)	Category B n (%)	Category C n (%)	*p*-Value
**Protocol adherence**					
	Prescribed according to MOH/hospital protocols	19,753 (62.5)	21,205 (53.5)	27,564 (57.5)	<0.001
	Not according to protocol	1735 (5.5)	3705 (9.3)	4310 (9.0)	
	Diagnosis outside protocol indications	10,122 (32.0)	14,744 (37.2)	16,040 (33.5)	
**Appropriateness of initial regimen**					
	Appropriate regimen	22,124 (70.0)	25,704 (64.8)	35,375 (73.8)	<0.001
	Inappropriate regimen	9486 (30.0)	13,950 (35.2)	12,539 (26.2)	
**Therapy management**					
	Appropriate management	13,480 (62.3)	17,377 (68.6)	20,239 (74.2)	<0.001
	Inappropriate management	8170 (37.7)	7958 (31.4)	7041 (25.8)	
**Therapeutic drug monitoring (Vancomycin/Aminoglycosides)**					
	TDM requested	3070 (41.4)	2783 (44.1)	2634 (50.2)	<0.001
	TDM not requested	4196 (56.6)	3423 (54.2)	2516 (47.9)	
	Not required	144 (1.9)	104 (1.6)	101 (1.9)	
**Physician response to ASP recommendations**					
	Accepted	22,172 (70.1)	26,585 (67.0)	26,321 (54.9)	<0.001
	Not accepted	839 (2.7)	2215 (5.6)	2529 (5.3)	
	No intervention recommended	8599 (27.2)	10,854 (27.4)	19,064 (39.8)	

Values are number (n) and percentage (%) within each hospital category. Analyses use the 119,178 records with recorded hospital category (category A, n = 31,610; category B, n = 39,654; category C, n = 47,914). Row denominators vary by variable because of variable-specific missingness. Protocol adherence in this table is computed on all reviewed prescriptions with a valid guideline response, including those with a diagnosis outside available protocol indications, whereas KPI 1 (Section 2.4) is restricted to prescriptions with an applicable MOH or hospital protocol; the two adherence figures are therefore not directly comparable. Physician acceptance percentages are computed on the full stratified subset within each category; the acceptance rate restricted to cases with an active intervention recommended (i.e., excluding “no intervention recommended”) is reported in the Amendment 4 narrative and is consistent with the KPI 5 acceptance rates in Section 2.8 (category A 96.4%; category B 92.3%; category C 91.2%). *p*-values are derived from chi-square tests of independence comparing distributions across category A, category B, and category C. Hospital categories reflect ASP capacity: category A, full ASP team with microbiology laboratory; category B, partial ASP core-team capacity; category C, no dedicated ASP team. ASP: antimicrobial stewardship programme; MOH: Ministry of Health; TDM: therapeutic drug monitoring.

**Table 4:** Protocol adherence to MOH/hospital antimicrobial guidelines stratified by demographic, institutional, clinical, and therapeutic characteristics (N = 81,995).

			N (%)	Adherent n (%)	Non-Adherent n (%)	*p*-Value
**Year of Data Collection**						
	2021		14,651 (17.9%)	12,872 (87.9%)	1779 (12.1%)	<0.001
	2022		25,259 (30.8%)	23,245 (92.0%)	2014 (8.0%)	
	2023		26,765 (32.6%)	22,210 (83.0%)	4555 (17.0%)	
	2024		15,320 (18.7%)	13,086 (85.4%)	2234 (14.6%)	
**Hospital Category**						
	A		21,488 (27.5%)	19,753 (91.9%)	1735 (8.1%)	<0.001
	B		24,910 (31.8%)	21,205 (85.1%)	3705 (14.9%)	
	C		31,874 (40.7%)	27,564 (86.5%)	4310 (13.5%)	
**Health Cluster**						
	**Central Region**		19,583 (23.9%)	16,549 (84.5%)	3034 (15.5%)	<0.001
		Riyadh	13,046 (15.9%)	11,227 (86.1%)	1819 (13.9%)	
		Al-Qassim	6537 (8.0%)	5322 (81.4%)	1215 (18.6%)	
	**Eastern Region**		9074 (11.1%)	7430 (81.9%)	1644 (18.1%)	
		Eastern Province	1838 (2.2%)	1578 (85.9%)	260 (14.1%)	
		Al-Ahsa	5705 (7.0%)	4810 (84.3%)	895 (15.7%)	
		Hafar AlBatin	1531 (1.9%)	1042 (68.1%)	489 (31.9%)	
	**Western Region**		23,623 (28.8%)	21,723 (92.0%)	1900 (8.0%)	
		Makkah	12,163 (14.8%)	11,522 (94.7%)	641 (5.3%)	
		Madinah	2704 (3.3%)	2225 (82.3%)	479 (17.7%)	
		Jeddah	6760 (8.2%)	6174 (91.3%)	586 (8.7%)	
		Taif	1996 (2.4%)	1802 (90.3%)	194 (9.7%)	
	**Northern Region**		13,034 (15.9%)	11,944 (91.6%)	1090 (8.4%)	
		Hail	5554 (6.8%)	5198 (93.6%)	356 (6.4%)	
		Tabuk	2604 (3.2%)	2340 (89.9%)	264 (10.1%)	
		Al-Jouf	3169 (3.9%)	3042 (96.0%)	127 (4.0%)	
		Northern Borders	1707 (2.1%)	1364 (79.9%)	343 (20.1%)	
	**Southern Region**		16,679 (20.3%)	13,765 (82.5%)	2914 (17.5%)	
		Aseer	4697 (5.7%)	3459 (73.6%)	1238 (26.4%)	
		Najran	1848 (2.3%)	1587 (85.9%)	261 (14.1%)	
		Jazan	2876 (3.5%)	2442 (84.9%)	434 (15.1%)	
		Al-Baha	7258 (8.9%)	6277 (86.5%)	981 (13.5%)	
**Clinical setting**						
	Ward class—non-critical care		46,451 (56.7%)	39,744 (85.6%)	6707 (14.4%)	<0.001
	Ward class—critical care		35,544 (43.3%)	31,669 (89.1%)	3875 (10.9%)	
**Infection syndrome**						
	Pneumonia and Respiratory Infections		38,376 (46.8%)	34,122 (88.9%)	4254 (11.1%)	<0.001
	Skin and Soft Tissue Infections		8379 (10.2%)	7202 (86.0%)	1177 (14.0%)	0.001
	Surgical Site Infection (SSI)		1332 (1.6%)	1089 (81.8%)	243 (18.2%)	<0.001
	Abdominal and GI Infection		7133 (8.7%)	6204 (87.0%)	929 (13.0%)	0.755
	Bloodstream and Systemic Infection		15,628 (19.1%)	13,870 (88.8%)	1758 (11.2%)	<0.001
	Bone and Joint Infections		611 (0.7%)	512 (83.8%)	99 (16.2%)	0.015
	Central Nervous System (CNS)		2206 (2.7%)	1990 (90.2%)	216 (9.8%)	<0.001
	Ear, Eye and Throat		56 (0.1%)	46 (82.1%)	10 (17.9%)	0.269
	Fungal and Parasitic		109 (0.1%)	94 (86.2%)	15 (13.8%)	0.79
	Genitourinary and STI		8733 (10.6%)	7587 (86.9%)	1146 (13.1%)	0.522
	Other Infectious Conditions		7895 (9.6%)	6283 (79.6%)	1612 (20.4%)	<0.001
**Indication for antibiotics**						
	Empiric therapy		52,851 (64.5%)	45,539 (86.2%)	7312 (13.8%)	<0.001
	Culture-guided therapy		21,755 (26.5%)	19,738 (90.7%)	2017 (9.3%)	
	Surgical prophylaxis		7389 (9.0%)	6136 (83.0%)	1253 (17.0%)	
**Therapy approach**						
	Monotherapy (1 antibiotic)		69,140 (84.4%)	60,949 (88.2%)	8191 (11.8%)	<0.001
	Combination therapy (≥2 antibiotics)		12,747 (15.6%)	10,373 (81.4%)	2374 (18.6%)	

Values are presented as n (%), with percentages calculated within rows. Protocol adherence refers to antimicrobial prescriptions consistent with MOH or hospital guidelines; analyses exclude cases classified as diagnoses outside guideline indications. Infection syndromes were analyzed as binary variables (yes/no); only “Yes” categories are shown, and categories are not mutually exclusive. *p*-values were derived from Pearson’s χ^2^ test (two-sided); *p* < 0.05 was considered statistically significant. For categorical variables with more than two levels, *p*-values reflect the overall group comparison; no single reference category is designated in this descriptive table.

**Table 5:** Restricted-antibiotic dispensing compliance among category A and B hospitals stratified by temporal, institutional, clinical, and therapeutic characteristics (N = 32,676).

			N (%)	Compliant n (%)	Non-Compliant n (%)	*p*-Value
**Hospital Category**						
	A		14,928 (45.7%)	13,537 (90.7%)	1391 (9.3%)	**<0.001**
	B		17,748 (54.3%)	15,080 (85.0%)	2668 (15.0%)	
**Year of Data Collection**						
	2021		8610 (26.3%)	7541 (87.6%)	1069 (12.4%)	**<0.001**
	2022		6173 (18.9%)	5601 (90.7%)	572 (9.3%)	
	2023		13,310 (40.7%)	11,286 (84.8%)	2024 (15.2%)	
	2024		4583 (14.0%)	4189 (91.4%)	394 (8.6%)	
**Health Cluster**						
	**Central Region**		4288 (13.1%)	3242 (75.6%)	1046 (24.4%)	**<0.001**
		Riyadh	3012 (9.2%)	2508 (83.3%)	504 (16.7%)	
		Al-Qassim	1276 (3.9%)	734 (57.5%)	542 (42.5%)	
	**Eastern Region**		4169 (12.8%)	3549 (85.1%)	620 (14.9%)	
		Eastern Province	1540 (4.7%)	1465 (95.1%)	75 (4.9%)	
		Al-Ahsa	2429 (7.4%)	1999 (82.3%)	430 (17.7%)	
		Hafar AlBatin	200 (0.6%)	85 (42.5%)	115 (57.5%)	
	**Western Region**		14,303 (43.8%)	13,325 (93.2%)	978 (6.8%)	
		Makkah	7160 (21.9%)	6995 (97.7%)	165 (2.3%)	
		Madinah	966 (3.0%)	862 (89.2%)	104 (10.8%)	
		Jeddah	5358 (16.4%)	4770 (89.0%)	588 (11.0%)	
		Taif	819 (2.5%)	698 (85.2%)	121 (14.8%)	
	**Northern Region**		4484 (13.7%)	4143 (92.4%)	341 (7.6%)	
		Hail	3194 (9.8%)	3086 (96.6%)	108 (3.4%)	
		Tabuk	677 (2.1%)	600 (88.6%)	77 (11.4%)	
		Al-Jouf	613 (1.9%)	457 (74.6%)	156 (25.4%)	
	**Southern Region**		5432 (16.6%)	4358 (80.2%)	1074 (19.8%)	
		Aseer	1632 (5.0%)	1088 (66.7%)	544 (33.3%)	
		Najran	740 (2.3%)	567 (76.6%)	173 (23.4%)	
		Jazan	422 (1.3%)	410 (97.2%)	12 (2.8%)	
		Al-Baha	2638 (8.1%)	2293 (86.9%)	345 (13.1%)	
**Clinical setting**						
	Ward class—non-critical care		14,409 (44.1%)	12,239 (84.9%)	2170 (15.1%)	**<0.001**
	Ward class—critical care		18,267 (55.9%)	16,378 (89.7%)	1889 (10.3%)	
**Infection syndrome**						
	Pneumonia and Respiratory Infections		11,816 (36.2%)	10,530 (89.1%)	1286 (10.9%)	**<0.001**
	Skin and Soft Tissue Infections		4068 (12.5%)	3587 (88.2%)	481 (11.8%)	0.217
	Surgical Site Infection (SSI)		467 (1.4%)	396 (84.8%)	71 (15.2%)	0.066
	Abdominal and GI Infection		3305 (10.1%)	2926 (88.5%)	379 (11.5%)	0.079
	Bloodstream and Systemic Infection		10,638 (32.5%)	9609 (90.3%)	1029 (9.7%)	**<0.001**
	Bone and Joint Infections		399 (1.2%)	353 (88.5%)	46 (11.5%)	0.586
	Central Nervous System (CNS)		1647 (5.0%)	1455 (88.3%)	192 (11.7%)	0.334
	Ear, Eye and Throat		17 (0.1%)	13 (76.5%)	4 (23.5%)	0.165
	Fungal and Parasitic		48 (0.1%)	43 (89.6%)	5 (10.4%)	0.673
	Genitourinary and STI		3265 (10.0%)	2898 (88.8%)	367 (11.2%)	**0.031**
	Other Infectious Conditions		1564 (4.8%)	972 (62.1%)	592 (37.9%)	**<0.001**
**Indication for antibiotics**						
	Empiric therapy		18,148 (55.5%)	15,522 (85.5%)	2626 (14.5%)	**<0.001**
	Culture-guided therapy		13,255 (40.6%)	12,248 (92.4%)	1007 (7.6%)	
	Surgical prophylaxis		1273 (3.9%)	847 (66.5%)	426 (33.5%)	
**Therapy approach**						
	Monotherapy (1 antibiotic)		27,268 (83.8%)	24,222 (88.8%)	3046 (11.2%)	**<0.001**
	Combination therapy (≥2 antibiotics)		5276 (16.2%)	4267 (80.9%)	1009 (19.1%)	

Analysis includes only category A and B hospitals and restricted-antibiotic prescriptions. Compliance is defined as restricted antibiotics dispensed after antimicrobial stewardship programme (ASP) approval. Infection syndromes were analyzed as binary variables (yes/no), and only “Yes” categories are presented; categories are not mutually exclusive. Analyses were conducted on available cases for each variable. *p*-values were derived from Pearson’s χ^2^ test (two-sided); *p* < 0.05 was considered statistically significant. For categorical variables with more than two levels, *p*-values reflect the overall group comparison. Northern Borders cluster is not displayed because no category A or B hospitals contributed restricted-antibiotic prescription data from this cluster.

**Table 6:** Antimicrobial stewardship interventions among reviewed antibiotic prescriptions.

ASP Interventions		N	%
	Optimize the therapy	27,709	22.3
	Dose corrected or adjusted	23,925	19.2
	Decide the management plan	21,948	17.6
	Request the culture	20,274	16.3
	Stop the antibiotic	19,601	15.8
	Information to nurse about medication administration	18,399	14.8
	Therapeutic drug monitoring	14,241	11.4
	Escalation of therapy	13,482	10.8
	Drug–drug interaction management	12,684	10.2
	Request laboratory investigations	12,504	10.0
	Change frequency	10,969	8.8
	De-escalation of therapy	9978	8.0
	Change duration	8512	6.8
	Change route of administration (IV→PO)	3799	3.1
	Continue the same (no intervention—appropriate regimen)	44,014	35.4

Values are presented as number (N) and percentage (%) of all reviewed antibiotic prescriptions (N = 124,452). ASP interventions represent actions taken by the antimicrobial stewardship team during review. Each reviewed case may have had more than one intervention recorded; therefore, individual percentages are not mutually exclusive, and the column total exceeds 100%. “Continue the same (no intervention—appropriate regimen)” denotes cases in which the existing regimen was reviewed and deemed appropriate without modification and is not counted as an active stewardship intervention for KPI 3 purposes.

**Table 7:** Annual trends in antimicrobial stewardship interventions, including de-escalation, antibiotic discontinuation, intravenous-to-oral conversion, and composite stewardship optimization rate among reviewed prescriptions in Saudi MOH hospitals, 2021–2024.

	2021 n (%)	2022 n (%)	2023 n (%)	2024 n (%)	*p*-Value
De-escalation	2110 (8.8%)	2050 (8.0%)	4199 (8.7%)	1619 (6.1%)	<0.001
Antibiotic discontinuation	4199 (17.6%)	4117 (16.0%)	7773 (16.1%)	3512 (13.2%)	<0.001
IV-to-oral conversion	471 (2.0%)	678 (2.6%)	1789 (3.7%)	861 (3.2%)	<0.001
Combined Stewardship Optimization Rate	5624 (23.5%)	5550 (21.6%)	11,080 (23.0%)	5185 (19.5%)	<0.001
Total reviewed	23,947 (19.2%)	25,730 (20.7%)	48,174 (38.7%)	26,601 (21.4%)	-

Values are presented as n (%). Percentages are calculated within each year using the total number of reviewed antimicrobial prescriptions as the denominator. De-escalation refers to narrowing or reducing antimicrobial spectrum; antibiotic discontinuation refers to cessation of therapy when no longer clinically indicated; intravenous-to-oral conversion refers to switching from parenteral to oral therapy when clinically appropriate. The combined stewardship optimization rate represents the proportion of prescriptions in which at least one of these three KPI 4 interventions was recorded and does not reflect total ASP intervention activity. *p*-values were derived from Pearson’s χ^2^ test (two-sided); *p* < 0.05 was considered statistically significant.

**Table 8:** Physician acceptance of ASP interventions stratified by temporal, institutional, and clinical characteristics (N = 84,747).

		N (%)	Accepted n (%)	Not Accepted n (%)	*p*-Value
**Year of Data Collection**					
	2021	17,147 (20.2%)	15,977 (93.2%)	1170 (6.8%)	<0.001
	2022	16,587 (19.6%)	14,887 (89.8%)	1700 (10.2%)	
	2023	33,206 (39.2%)	31,037 (93.5%)	2169 (6.5%)	
	2024	17,807 (21.0%)	16,886 (94.8%)	921 (5.2%)	
**Hospital Category**					
	A	23,011 (28.5%)	22,172 (96.4%)	839 (3.6%)	<0.001
	B	28,800 (35.7%)	26,585 (92.3%)	2215 (7.7%)	
	C	28,850 (35.8%)	26,321 (91.2%)	2529 (8.8%)	
**Clinical setting**					
	Ward class—non-critical care	43,692 (51.6%)	39,279 (89.9%)	4413 (10.1%)	<0.001
	Ward class—critical care	41,055 (48.4%)	39,508 (96.2%)	1547 (3.8%)	
**Infection syndrome**					
	Pneumonia and Respiratory Infections	30,912 (36.5%)	28,970 (93.7%)	1942 (6.3%)	<0.001
	Skin and Soft Tissue Infections	10,702 (12.6%)	9784 (91.4%)	918 (8.6%)	<0.001
	Surgical Site Infection (SSI)	1478 (1.7%)	1317 (89.1%)	161 (10.9%)	<0.001
	Abdominal and GI Infection	7027 (8.3%)	6475 (92.1%)	552 (7.9%)	0.005
	Bloodstream and Systemic Infection	23,396 (27.6%)	22,566 (96.5%)	830 (3.5%)	<0.001
	Bone and Joint Infections	1104 (1.3%)	1028 (93.1%)	76 (6.9%)	0.846
	Central Nervous System (CNS)	3906 (4.6%)	3741 (95.8%)	165 (4.2%)	<0.001
	Ear, Eye and Throat	103 (0.1%)	96 (93.2%)	7 (6.8%)	0.925
	Fungal and Parasitic	266 (0.3%)	256 (96.2%)	10 (3.8%)	0.037
	Genitourinary and STI	6753 (8.0%)	6295 (93.2%)	458 (6.8%)	0.401
	Other Infectious Conditions	7565 (8.9%)	6228 (82.3%)	1337 (17.7%)	<0.001
**Indication for antibiotics**					
	Empiric therapy	52,650 (62.1%)	48,940 (93.0%)	3710 (7.0%)	<0.001
	Culture-guided therapy	25,045 (29.6%)	24,048 (96.0%)	997 (4.0%)	
	Surgical prophylaxis	7052 (8.3%)	5799 (82.2%)	1253 (17.8%)	
**Therapy approach**					
	Monotherapy (1 antibiotic)	72,573 (85.8%)	67,758 (93.4%)	4815 (6.6%)	<0.001
	Combination therapy (≥2 antibiotics)	12,009 (14.2%)	10,873 (90.5%)	1136 (9.5%)	

Values are presented as n (%), with percentages calculated within rows. Physician acceptance was evaluated among 84,747 cases with an active ASP recommendation, excluding 39,705 cases (31.9%) where no intervention was required. Infection syndromes were analyzed as binary variables (yes/no); only “Yes” categories are shown, and categories are not mutually exclusive. Analyses were conducted on available cases for each variable. *p*-values were derived from Pearson’s χ^2^ test (two-sided); *p* < 0.05 was considered statistically significant. For categorical variables with more than two levels, *p*-values reflect the overall group comparison.

**Table 9:** Appropriate antimicrobial therapy management stratified by temporal, institutional, clinical, and therapeutic characteristics (N = 77,588).

			N (%)	Appropriate n (%)	Inappropriate n (%)	*p*-Value
**Year of Data Collection**						
	2021		17,146 (22.1%)	11,139 (65.0%)	6007 (35.0%)	<0.001
	2022		25,672 (33.1%)	18,237 (71.0%)	7435 (29.0%)	
	2023		25,573 (33.0%)	17,569 (68.7%)	8004 (31.3%)	
	2024		9197 (11.9%)	6013 (65.4%)	3184 (34.6%)	
**Hospital Category**						
	A		21,650 (29.2%)	13,480 (62.3%)	8170 (37.7%)	<0.001
	B		25,335 (34.1%)	17,377 (68.6%)	7958 (31.4%)	
	C		27,280 (36.7%)	20,239 (74.2%)	7041 (25.8%)	
**Health Cluster**						
	**Central Region**		16,814 (21.7%)	13,397 (79.7%)	3417 (20.3%)	<0.001
		Riyadh	9658 (12.4%)	7582 (78.5%)	2076 (21.5%)	
		Al-Qassim	7156 (9.2%)	5815 (81.3%)	1341 (18.7%)	
	**Eastern Region**		10,278 (13.2%)	7397 (72.0%)	2881 (28.0%)	
		Eastern Province	2487 (3.2%)	1861 (74.8%)	626 (25.2%)	
		Al-Ahsa	5932 (7.6%)	4676 (78.8%)	1256 (21.2%)	
		Hafar AlBatin	1859 (2.4%)	860 (46.3%)	999 (53.7%)	
	**Western Region**		23,318 (30.1%)	13,345 (57.2%)	9973 (42.8%)	
		Makkah	11,020 (14.2%)	6049 (54.9%)	4971 (45.1%)	
		Madinah	2783 (3.6%)	1509 (54.2%)	1274 (45.8%)	
		Jeddah	8177 (10.5%)	5314 (65.0%)	2863 (35.0%)	
		Taif	1338 (1.7%)	473 (35.4%)	865 (64.6%)	
	**Northern Region**		12,206 (15.7%)	8231 (67.4%)	3975 (32.6%)	
		Hail	4194 (5.4%)	2115 (50.4%)	2079 (49.6%)	
		Tabuk	2202 (2.8%)	1609 (73.1%)	593 (26.9%)	
		Al-Jouf	3825 (4.9%)	3339 (87.3%)	486 (12.7%)	
		Northern Borders	1985 (2.6%)	1168 (58.8%)	817 (41.2%)	
	**Southern Region**		14,971 (19.3%)	10,587 (70.7%)	4384 (29.3%)	
		Aseer	3631 (4.7%)	2049 (56.4%)	1582 (43.6%)	
		Najran	927 (1.2%)	669 (72.2%)	258 (27.8%)	
		Jazan	3073 (4.0%)	1974 (64.2%)	1099 (35.8%)	
		Al-Baha	7340 (9.5%)	5895 (80.3%)	1445 (19.7%)	
**Clinical setting**						
	Ward class—non-critical care		43,429 (56.0%)	31,363 (72.2%)	12,066 (27.8%)	<0.001
	Ward class—critical care		34,159 (44.0%)	21,595 (63.2%)	12,564 (36.8%)	
**Infection syndrome**						
	Pneumonia and Respiratory Infections		29,458 (38.0%)	21,267 (72.2%)	8191 (27.8%)	<0.001
	Skin and Soft Tissue Infections		9429 (12.1%)	6289 (66.7%)	3140 (33.3%)	0.001
	Surgical Site Infection (SSI)		1171 (1.5%)	678 (57.9%)	493 (42.1%)	<0.001
	Abdominal and GI Infection		6779 (8.7%)	4419 (65.2%)	2360 (34.8%)	<0.001
	Bloodstream and Systemic Infection		19,248 (24.8%)	13,067 (67.9%)	6181 (32.1%)	0.206
	Bone and Joint Infections		901 (1.2%)	580 (64.4%)	321 (35.6%)	0.012
	Central Nervous System (CNS)		3249 (4.2%)	2020 (62.2%)	1229 (37.8%)	<0.001
	Ear, Eye and Throat		36 (0.05%)	28 (77.8%)	8 (22.2%)	0.22
	Fungal and Parasitic		182 (0.2%)	127 (69.8%)	55 (30.2%)	0.658
	Genitourinary and STI		6192 (8.0%)	4133 (66.7%)	2059 (33.3%)	0.008
	Other Infectious Conditions		8585 (11.1%)	5219 (60.8%)	3366 (39.2%)	<0.001
**Indication for antibiotics**						
	Empiric therapy		47,679 (61.5%)	32,248 (67.6%)	15,431 (32.4%)	<0.001
	Culture-guided therapy		21,737 (28.0%)	15,631 (71.9%)	6106 (28.1%)	
	Surgical prophylaxis		8172 (10.5%)	5079 (62.2%)	3093 (37.8%)	
**Therapy approach**						
	Monotherapy (1 antibiotic)		64,447 (83.1%)	44,750 (69.4%)	19,697 (30.6%)	<0.001
	Combination therapy (≥2 antibiotics)		12,984 (16.7%)	8098 (62.4%)	4886 (37.6%)	

Values are presented as n (%), with percentages calculated within rows. Therapy management data were available for 77,588 of 124,452 reviewed cases (62.3%); the remaining cases had missing therapy management documentation and were excluded from this analysis. Appropriate therapy management reflects the ASP team’s documented assessment at the time of review and should not be interpreted as an independently verified measure of prescribing quality; findings may be influenced by stewardship team expertise, documentation intensity, and case complexity. Infection syndromes are not mutually exclusive. *p*-values were derived from Pearson’s χ^2^ test (two-sided); *p* < 0.05 was considered statistically significant. For categorical variables with more than two levels, *p*-values reflect the overall group comparison.

**Table 10:** Multivariable binary logistic regression analysis of factors independently associated with guideline non-adherence (N = 78,164) and physician non-acceptance of ASP recommendations (N = 80,496), Saudi MOH hospitals, 2021–2024.

		Guideline Non-Adherence			Physician Non-Acceptance of ASP Recommendations		
		Adjusted OR	95% CI	*p*-Value	Adjusted OR	95% CI	*p*-Value
**Year of Data Collection**				<0.001			<0.001
	2021	1			1		
	2022	0.509	0.474–0.546	<0.001	1.337	1.233–1.451	<0.001
	2023	1.272	1.197–1.352	<0.001	0.796	0.737–0.859	<0.001
	2024	1.277	1.191–1.370	<0.001	0.633	0.577–0.696	<0.001
**Hospital Category**				<0.001			<0.001
	A	1			1		
	B	1.819	1.711–1.935	<0.001	1.788	1.645–1.944	<0.001
	C	1.549	1.458–1.646	<0.001	2.019	1.860–2.192	<0.001
**Clinical setting**				<0.001			<0.001
	Ward class—critical care	1			1		
	Ward class—non-critical care	1.349	1.287–1.414	<0.001	2.28	2.137–2.434	<0.001
**Indication for antibiotics**				<0.001			<0.001
	Surgical prophylaxis	1			1		
	Empiric therapy	0.748	0.696–0.803	<0.001	0.463	0.430–0.499	<0.001
	Culture-guided therapy	0.492	0.453–0.535	<0.001	0.29	0.264–0.319	<0.001
**Therapy approach**				<0.001			<0.001
	Monotherapy (1 antibiotic)	1			1		
	Combination therapy (≥2 antibiotics)	1.702	1.608–1.802	<0.001	1.418	1.316–1.529	<0.001

Adjusted odds ratios (aORs) and 95% confidence intervals (CIs) were derived from multivariable binary logistic regression using the enter method. Reference categories were 2021, hospital category A, critical care ward, surgical prophylaxis, and monotherapy. Cases classified as outside guideline indications were excluded from the non-adherence model; cases without an active ASP recommendation were excluded from the non-acceptance model. Both models were statistically significant (*p* < 0.001). aOR, adjusted odds ratio; ASP, antimicrobial stewardship programme; CI, confidence interval.

**Table 11:** Hospital-level use of monitored Watch and reserve antimicrobial agents, expressed as defined daily doses (DDD) per 100 available bed-days, by year and hospital ASP category.

Year and Group	Units (n)	Total DDD	Available Bed-Days	DDD/100 Available Bed-Days (Pooled)	Median (IQR) Across Hospitals	Reserve DDD/100 Available Bed-Days	*p*-Value
**2022—All hospitals**	**57**	**683,479**	**6,635,700**	**10.30**	**5.90 (3.14–13.28)**	**0.655**	**0.042**
Category A	13	242,392	1,918,440	12.63	11.32 (5.62–18.12)	0.867	
Category B	19	280,792	2,384,180	11.78	6.79 (4.48–15.71)	0.739	
Category C	25	160,294	2,333,080	6.87	4.51 (2.83–8.86)	0.396	
**2023—All hospitals**	**57**	**738,777**	**6,635,700**	**11.13**	**7.52 (4.00–12.56)**	**0.630**	**0.008**
Category A	13	347,972	1,918,440	18.14	12.56 (10.22–23.11)	1.045	
Category B	19	207,765	2,384,180	8.71	5.87 (4.80–9.87)	0.441	
Category C	25	183,041	2,333,080	7.85	4.90 (3.15–10.08)	0.483	
**2024—All hospitals**	**56**	**697,796**	**6,750,626**	**10.34**	**5.98 (3.04–11.14)**	**0.566**	**0.032**
Category A	14	322,443	2,154,276	14.97	10.55 (6.04–19.30)	0.746	
Category B	21	225,399	2,601,162	8.67	7.08 (3.06–9.19)	0.462	
Category C	21	149,954	1,995,188	7.52	3.80 (2.35–6.80)	0.507	

Antimicrobial-use returns were available from 60 separately reporting hospital units representing 58 of the 96 participating hospitals, although not all units reported in every year. The monitored agents were meropenem, imipenem–cilastatin, piperacillin–tazobactam, and vancomycin from the WHO AWaRe Watch group, and colistin from the reserve group. Access-group agents were not captured; therefore, the reported values do not represent total hospital antimicrobial use. Available bed-days were calculated as licenced bed capacity multiplied by the number of reporting days. DDD per 100 available bed-days should be interpreted as a capacity-adjusted measure and is expected to underestimate DDD per 100 occupied bed-days when occupancy is below 100%. The pooled rate was calculated as total DDD divided by total available bed-days and multiplied by 100, whereas the median and interquartile range describe the distribution of hospital-level rates. Two implausible agent-level values were excluded. Differences across categories A, B, and C were assessed using the Kruskal–Wallis test. ASP, antimicrobial stewardship programme; DDD, defined daily dose; IQR, interquartile range.

## Data Availability

The data supporting the findings of this study are available from the corresponding author upon reasonable request, subject to approval by the Saudi Ministry of Health and applicable data-governance requirements.

## Supplementary Materials

The following supporting information can be downloaded at https://www.mdpi.com/article/10.3390/antibiotics15080753/s1, Table S1. Characterisation of the 42,457 prescriptions classified as “diagnosis outside protocol indications” in the national ASP surveillance dataset, 2021–2024, including: (A) Primary infection syndrome distribution; (B) Eight most frequent unambiguously outside-protocol individual diagnoses; and (C) Indication for antibiotic therapy.

## Abbreviations

The following abbreviations are used in this manuscript:

AMR	Antimicrobial resistance
ASP	Antimicrobial stewardship programme
CRE	Carbapenem-resistant Enterobacterales
DDD	Defined daily dose
ESBL	Extended-spectrum beta-lactamase
IDSA	Infectious Diseases Society of America
KPI	Key performance indicator
MDR	Multidrug-resistant
MOH	Ministry of Health
MRSA	Methicillin-resistant *Staphylococcus aureus*
NDM	New Delhi metallo-beta-lactamase
OXA-48	Oxacillinase-48
SHEA	Society for Healthcare Epidemiology of America
SSI	Surgical site infection
STI	Sexually transmitted infection
TDM	Therapeutic drug monitoring
VRE	Vancomycin-resistant *Enterococcus*
XDR	Extensively drug-resistant
