# Supplementary material for: National Antimicrobial Stewardship Performance and Antimicrobial Use in Saudi Ministry of Health Hospitals: A Four-Year Multicenter Surveillance Analysis, 2021–2024
Source: Antibiotics (Basel). 2026 Aug 4;15(8):753. doi: 10.3390/antibiotics15080753 (PMC13509192; doi:10.3390/antibiotics15080753)
Supplement: Supplementary file 1 [file antibiotics-15-00753-s001.zip › antibiotics-4387241-supplementary.pdf]

Supplementary Table S1. Characterisation of the 42,457 prescriptions classified as "diagnosis outside protocol indications" in the national ASP surveillance dataset, 2021–2024.

Panel A. Primary infection syndrome distribution

Primary syndrome	N	%
Bloodstream and systemic infections	12,481	29.4
Other infectious conditions	8,755	20.6
Pneumonia and respiratory infections	5,986	14.1
Skin and soft tissue infections	5,207	12.3
Abdominal and gastrointestinal infections	3,356	7.9
Multiple primary syndromes recorded simultaneously	2,521	5.9
Central nervous system (CNS) infections	2,386	5.6
Genitourinary and sexually transmitted infections	880	2.1
Bone and joint infections	658	1.5
Fungal and parasitic infections	227	0.5

Panel B. Eight most frequent unambiguously outside-protocol individual diagnoses

Specific Diagnosis	N	%
Sepsis	6,182	14.6
Septic shock	2,430	5.7
Bloodstream infection	2,175	5.1
Upper respiratory tract infection	2,063	4.9
Abdominal infection	1,277	3
Lower respiratory tract infection (other than CAP, VAP, or HAP)	1,267	3
Central nervous system infection, unspecified	1,207	2.8
Other soft skin infection	1,093	2.6
Other diagnoses	24,763	58.3
**Total**	**42,457**	**100**

Panel C. Indication for antibiotic therapy

Indication for Antibiotics	N	%
Empiric therapy	23,204	54.7
Culture-guided therapy	10,186	24
Surgical prophylaxis	9,067	21.4
**Total**	42,457	100

Values are number (N) and percentage (%) of the 42,457 prescriptions with a diagnosis outside available Ministry of Health or hospital antimicrobial protocol indications, identified in the national ASP surveillance dataset for 2021–2024. Percentages in each panel are calculated on the full 42,457-case denominator. The five conditions with dedicated MOH/hospital antimicrobial protocols during the study period were pneumonia, urinary tract infection, cellulitis and skin/soft tissue infection, intra-abdominal infection, and surgical site infection; diagnoses not falling within these categories were classified as "diagnosis outside protocol indications". **Panel A** reports the primary infection syndrome; the "Multiple primary syndromes" row captures cases with more than one syndrome documented concurrently. **Panel B** lists the eight most frequently recorded unambiguously outside-protocol individual diagnoses (each with >1,000 cases); the "Other diagnoses" row aggregates all remaining free-text entries. **Panel C** reports the antibiotic indication; the 9,067 outside-protocol surgical prophylaxis cases reflect the fact that surgical site infection was added to the ASP form's protocol list only in 2023, and that surgical prophylaxis is a distinct perioperative activity governed by a separate national protocol not captured by the surveillance form (empirically, 98.6% of these cases did not carry the surgical site infection syndrome flag). ASP: antimicrobial stewardship programme; MOH: Ministry of Health; CAP: community-acquired pneumonia; VAP: ventilator-associated pneumonia; HAP: hospital-acquired pneumonia.
